# Illuminating the complex interplay of risk factors for depression symptoms within a large-scale US longitudinal cohort

**DOI:** 10.1007/s00127-026-03098-0

**Published:** 2026-05-05

**Authors:** Katherine N. Thompson, Baptiste Couvy-Duchesne, Sang Hong Lee, Rafael Geurgas, Yeongmi Jeong, Saranya Arirangan, Saul Newman, Felix C. Tropf, Robbee Wedow

**Affiliations:** 1 Department of Sociology, Purdue University, West Lafayette, IN US; 2 Institute for Molecular Bioscience, University of Queensland, Brisbane, Australia; 3 Paris Brain Institute - ICM, Sorbonne University, CNRS, Inria, AP-HP, Hopital de la Pitie Salpetriere, Paris, Inserm France; 4 Brain & Mental Health Program, QIMR Berghofer Medical Research Institute, Brisbane, Australia; 5 School of Biomedical Sciences, Queensland University of Technology, Brisbane, Australia; 6 Australian Centre for Precision Health, School of Public Health, College of Health , Adelaide University, Adelaide, Australia; 7 UniSA Allied Health and Human Performance, University of South Australia, Adelaide, Australia; 8 South Australian Health and Medical Research Institute, Adelaide, Australia; 9 Centre for Longitudinal Studies, University College London, London, UK; 10 Department of Medical and Molecular Genetics, Indiana University School of Medicine, Indianapolis, US

**Keywords:** Systems, Gene-environment interplay, Exposome, Longitudinal cohort, Social acceptance, Depression

## Abstract

**Purpose:**

Understanding depression susceptibility requires research to assimilate a vast and complex array of risk factors comprehensively. We quantify the influence of prominent individual-level and wider social environmental risk factors on symptom occurrence.

**Methods:**

We leverage data from the National Longitudinal Study of Adolescent to Adult Health (US) to integrate factors spanning biological (genetic), psychological (health, personality, positive cognition), social (family, peers, school, and neighbourhood), built (geocoded healthcare, education, religion, crime, poverty, political climate), and natural (geocoded population density, rainfall, and urbanicity) systems. We test their contribution to concurrent adolescent depression symptoms (W1), one year later (W2), and a decade later in early adulthood (W4) using relatedness-based linear mixed models (European-like ancestries only; *N* = 3,867).

**Results:**

First, a subset of 81 individual-level factors together explained 85.5% of the variance in concurrent adolescent depression symptoms, 68.4% one year later, and 52.5% in adulthood. When split by domains, positive cognition (feeling accepted and loved) contributed most in adolescence (W1 15.1% [SE = 4%]; W2 6.6% [2%]), and domains contributed equally in adulthood. Second, all 162 factors capturing interconnected genetic, psychological, social, built, and natural systems explained 80.5% in concurrent depression symptoms, 54.7% one year later, and 44.9% in adulthood. The psychological system (W1 38.5% [4%]; W2 21.9% [3%]; W4 7.7% [1%], psychological-genetic interaction (W1 26.7% [4%]; W2 25.1% [4%]; W4 12.4% [3%]), social-genetic interaction (W1 10.1% [3%]; W2 11.8% [3%]; W4 9.6% [3%]) explained most variance in depression symptoms at all ages.

**Conclusion:**

We provide a holistic understanding of depression risk, where feeling supported and accepted was most crucial, and emphasise the complexity of modelling the environment.

**Supplementary Information:**

The online version contains supplementary material available at https://doi.org/10.1007/s00127-026-03098-0.

## Introduction

Comprehending why people develop mental health disorders is central to our understanding of susceptibility. Depression is one of the leading causes of disability worldwide [1], and around 300 million people experience symptoms [2]. Decades of research have identified a plethora of risk factors for depression. However, many studies have focused on a handful of factors at one time, such as gender, socioeconomic status, stressful life events, family history, and genetic vulnerability [3–7]. Building on this work, there is increasing recognition to integrate these many factors to better capture the extent and complexity of the contexts that shape people’s experiences.

Three overarching challenges persist when researching risk factors for depression. First, despite increasing public health efforts and the acknowledgement of many determinants, it is difficult to conceptualise and test the influence of a myriad of factors. Depression is multifactorial; every part of a person’s surroundings (environment or exposome), where they are born, live, learn, work, and play are linked to depression [4]. It is difficult to comprehensively identify risk when the factors are as vast as they are complex. The social conditions in which people live influence depression and are manifested by powerful, money-driven institutions that produce inequality [8]. Individual-level factors are set within a wider-environment, thus inextricably linked to life cir­cumstances and societal structures. A growing body of research has begun to develop environment-wide methodological approaches, such as exposome-wide association studies on mental health outcomes [9, 10]. For example, one study mapped out the exposome of mental health by testing for individual associations with 294 environmental variables across 7 psychiatric traits [9]. Other work aimed to operationalise ecological systems theory by using latent class analysis to create a comprehensive view of adolescent family, peers, school, and neighbourhood contexts [11], and they found that adolescent social relationships were particularly important for depression. These large-scale data efforts support a perspective that depression can arise from many different systems, which are interconnected and depend on one another, both across and within systems [12–14]. Innovative models are needed to operationalise and bring together these systems and capture how they interact to increase depression risk.

Second, understanding depression risk requires a developmental lens. Extensive research has demonstrated the need for longitudinal designs as depression likely emerges from a dynamic interplay between factors that can change over time [15]. Detailed longitudinal cohort studies provide a valuable opportunity to both capture a wide range of factors and assess depression symptoms at multiple time points. Adolescence is a particularly critical period of development, marked by biological and social transitions, during which the onset of depression is typical [16, 17]. Developmental social-cognitive theorists have long conceptualised adolescence as a critical period for identity formation and self-definition [18]. A wide range of experiences in adolescence, such as maltreatment, family conflict, and socioeconomic deprivation, can disrupt emotion regulation, stress-response systems, and social-cognitive development and lead to increased depression risk [19]. As an example, the cumulative exposure to childhood adversities has been associated with early adulthood depression [20]. Distinguishing which factors are associated with concurrent, short-term, and long-term depression symptoms is essential for clarifying the developmental timing of risk.

Third, it is challenging to capture the interplay of genetic, social, familial, socioeconomic, and structural factors. A person’s experiences do not happen in isolation; factors are interconnected, combine, and interact to increase depression risk [21]. For example, stressful life events could have a stronger impact on depression among individuals with higher genetic risk (gene-environment interaction) [22, 23], and school support is reliant on government funding for that area [24]. Machine learning showed potential to analyse many factors, but psychiatric studies have limited success in predicting above chance, are heavily based on class imbalance, demonstrate overfitting, and do not adequately assess predictive performance [25–27]. However, there is a distinction between understanding the factors underlying depression (explanation) and machine learning predicting which individuals will or will not become depressed (prediction) [28]. Using relatedness matrices focuses on explanation by incorporating a large number of factors with small effects and assessing their contribution to the variance in a trait [29] and has been applied to genetic [30], gene methylation [31], neuroimaging [32], and one set of environmental data for adolescent mental health [33]. Using large-scale systems data to comprehensively distinguish which factors play a major role and the intricate ways they combine to increase depression risk is key to translate findings from holistic approaches to intervention efforts.

**Fig. 1:**
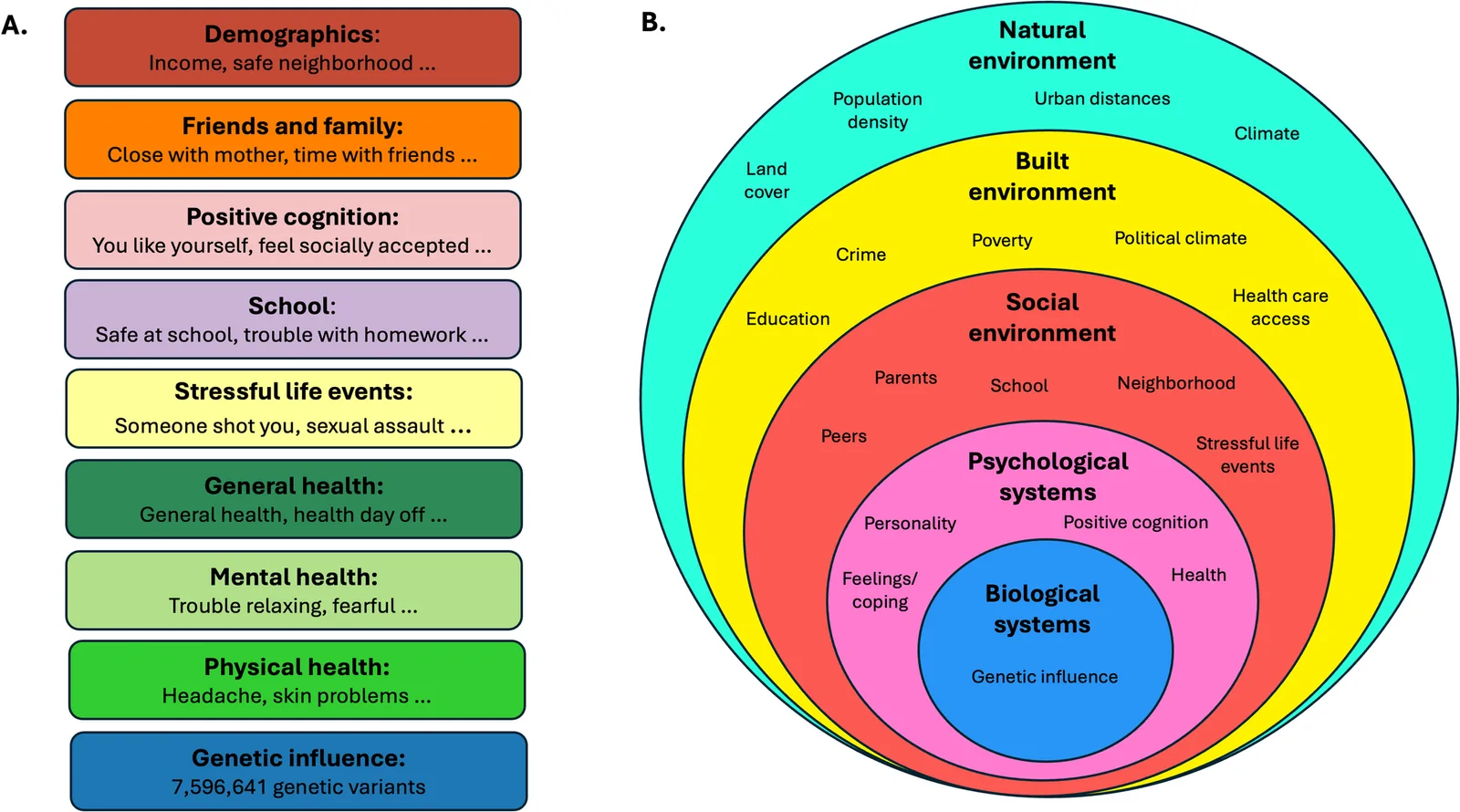
Visualisation of the depression risk factor domains and systems included in research question 1 and 2. Panel A shows research question 1: We identify individual-level risk factors commonly reported in the literature to be associated with depression symptoms that are measured in adolescence in Add Health. We organise the data into domains that reflect demographics, relationships with friends and family, physical health, mental health, general health, feelings about personal abilities and surroundings, which we term positive cognition, school experiences, stressful life events, and genetic influence. We aim to estimate the variance explained by these factors for depression symptoms concurrently in adolescence (age 12–20), one year later (age 13–21), and longitudinally in adulthood (age 24–32). Panel B shows research question 2: We extend the initial individual-level factors to include 162 variables measured at adolescence that encompass biological systems, psychological systems, social environment, the built environment, and the natural environment. We aim to model the contribution of each system, and the interaction between systems, to depression symptoms concurrently in adolescence and longitudinally in adulthood.

To address these challenges, we leverage data from the U.S. National Longitudinal Study of Adolescent to Adult Health (Add Health) to integrate a multitude of life factors in adolescence and assess how they contribute to concurrent, short-term (one year later), and long-term (over a decade later) depression symptoms. Our work innovatively applies a relatedness matrix-based approach to comprehensively incorporate and quantify the joint contribution of an array of individual, social, and structural factors to depression symptoms from adolescence into adulthood. We ask: (1) *How do prominent adolescent individual-level risk factors for depression contribute to concurrent and longitudinal symptom occurrence?*; (2) *To what extent does the wider environment contribute to depression symptoms?* (Fig. 1).

## Methods

### Sample

We used data from Add Health: a U.S. nationally representative cohort of people born between 1976 and 1982. The cohort profile is described elsewhere [34], and the full sample description is provided in **Supplement 1**. Briefly, we included social, behavioural, genetic, and contextual data collected at Wave 1 (W1; aged 12–21; *N* = 20,745), and depression symptom information at Wave 2 (W2; aged 13–22; *N* = 14,738), and Wave 4 (W4; aged 24–34; *N* = 15,701) to reflect early adulthood. Our initial sample consisted of 9,974 individuals with complete longitudinal data and 8,830 that passed genomic data quality control. Population stratification can confound whole-genome effects. To retain power and sample size, we therefore focused on the largest ancestry group of European-like ancestries (*N* = 5,729). We retained 9,247,076 variants with imputation INFO ≥ 0.10, missingness < 0.02, call rate ≥ 0.98, minor allele frequency ≥ 1%, and Hardy-Weinberg≥1e-06. The top 20 principal components were used to adjust for residual stratification. We randomly selected one individual from each related pair (pi-hat > 0.125). Our final sample included 3,867 unrelated people of European-like ancestries with non-missing depression symptom outcome data at Wave 1, 2, and 4 (53.10% female, Wave 1 mean age = 15.20 [12–19]; Wave 2 mean age = 16.11 [13–20]; Wave 4 mean age = 28.07 [24–33]).

## Measures

### Depression symptoms

The self-report Centre for Epidemiologic Studies-Depression scale (CES-D) was used to measure depression symptoms [35]. We created a reduced 10-item depression symptom sum score at each Wave (**Supplement 1 A**). The full CES-D score was also computed at Wave 1 and results for this score are also provided in the Supplement. Responses for each item were 0 (never), 1 (sometimes), 2 (a lot of the time), and 3 (most or all the time). Depression scores were centred and standardised, with a mean of 0 and a standard deviation of 1.

## Risk factors

Given the complexity and scope of the life experiences, characteristics, and surroundings associated with depression, we identified hundreds of risk and protective factors relevant to depression that were measured in Add Health at Wave 1, collected across multiple data sources (self-report, parent-report, and geocoded data). We split this data into two stages to reflect our two research questions. First, we used 81 *individual-level* depression risk factors based on two recent reviews [4, 8]. This includes interview and survey questions capturing demographics, social characteristics, health, school and family experiences, and traumatic events. Second, we substantially expanded these to reflect a systems perspective, including 162 factors. Using system levels of the ecological model [12], we structured these variables using distinct groupings [36] and terminology from the systems model of mental health and resilience [14]. These systems include biological systems (genetic data), psychological systems (37 self-report items on health, personality, positive cognition, and self-worth), social environment (49 self-report items on family, peers, school, and neighbourhood experiences), built environment (33 geocoded societal structural and political context variables on healthcare, education, labour force, religion, crime, poverty, political climate), and the natural environment (15 geocoded weather and location variables such as population density, rainfall, and urban distances). All risk factor variables were collected at Wave 1, therefore, we test how adolescent exposures are associated with concurrent (W1), short-term (W2), and long-term (W4) depression symptoms. Active missing data (reports of “don’t know” or “prefer not to answer” were included as responses and we used random-sample imputation to account for remaining missingness (**Supplement 1B & 1 C).**

## Statistical analyses

We examined how large-scale data collected in adolescence contribute to depression symptoms concurrently, and longitudinally in the short-term (one year later) and long-term (14 years later) using relatedness-matrix-based linear mixed models (LMM) with restricted maximum likelihood (REML). LMMs combine fixed effects and random effects to analyse data that has structured non-independence [29]. Typically, random effects are used to account for clustering. We extend this by using relatedness matrices to define the random effect covariance structure, which quantifies the degree of pairwise similarity between individuals on a set of variables, allowing us to estimate the variance explained in depression symptoms by environmental/genetic similarity in our sample, without discrete groupings. Therefore, LMMs estimate the portion of variance that can be attributed to similarity structures between individuals, as captured by the random effects. This contrasts with regression models using fixed-effects (R^2^), which instead estimate incremental individual-level variation that can be predicted by variables. Thus, estimates should be interpreted as descriptive variance components reflecting similarity structures in the sample, rather than individual-predictive metrics or causal effects of specific exposures.

To reduce multicollinearity and avoid biased variance component estimates [29], we transformed the data by multiplying by covariance-extracted eigenvectors. For our LMMs, we first constructed pairwise similarity (relatedness) matrices for each set of variables (genetic and environmental). Genetic relatedness matrices were computed using single-nucleotide polymorphism (SNP) data, analogous to SNP-based heritability. Environmental matrices were computed using the mtg2 software [37], whereby for every pair of individuals, a continuous value is constructed for how similar they were across variables. These matrices defined the random-effect covariance structures in the linear model, so that the random-effect variance represents variance in depression symptoms attributable to similarity patterns between individuals. Details provided in **Supplement 1D**.

We fit a comprehensive series of LMMs to decompose the variance in depression symptoms into contributions from genetic, environmental, and error components. We start with one broad environmental effect and a genetic effect, then split this into random effects that reflect variable domains. We then extend the data to create random effects that represent each system. Each set of models includes covariance [38] and interaction terms (Box 1) to assess the interplay between the random effects. We use likelihood ratio tests to assess model fit. Analyses were pre-registered (https://osf.io/akzhd/*)*, and code is on GitHub (https://github.com/knthompson26/findmedep*).*

Box 1 When we describe gene-environment interaction components, we are not modelling gene-environment interaction (GxE) in the traditional sense. GxE describes how genetic predispositions interact with or modify the influence of environmental exposures, and vice versa, such that genetic effects vary across different external environments. For example, in regression models that include polygenic score × exposure terms. Here, we refer to an interaction between the similarity of individuals on a set of characteristics and experiences and their similarity in genetic data as captured by a variance component defined by the Hadamard (element-wise) product of the genetic relatedness matrix and the environmental similarity matrix. This interaction, therefore, reflects excess phenotypic covariance among individuals who are jointly similar genetically and environmentally, rather than the moderation of individual-level genetic effects.

## Results

### How do prominent individual-level risk factors for depression contribute to symptom occurrence?

#### Broad environmental and genetic influences on depression symptoms

**Fig. 2:**
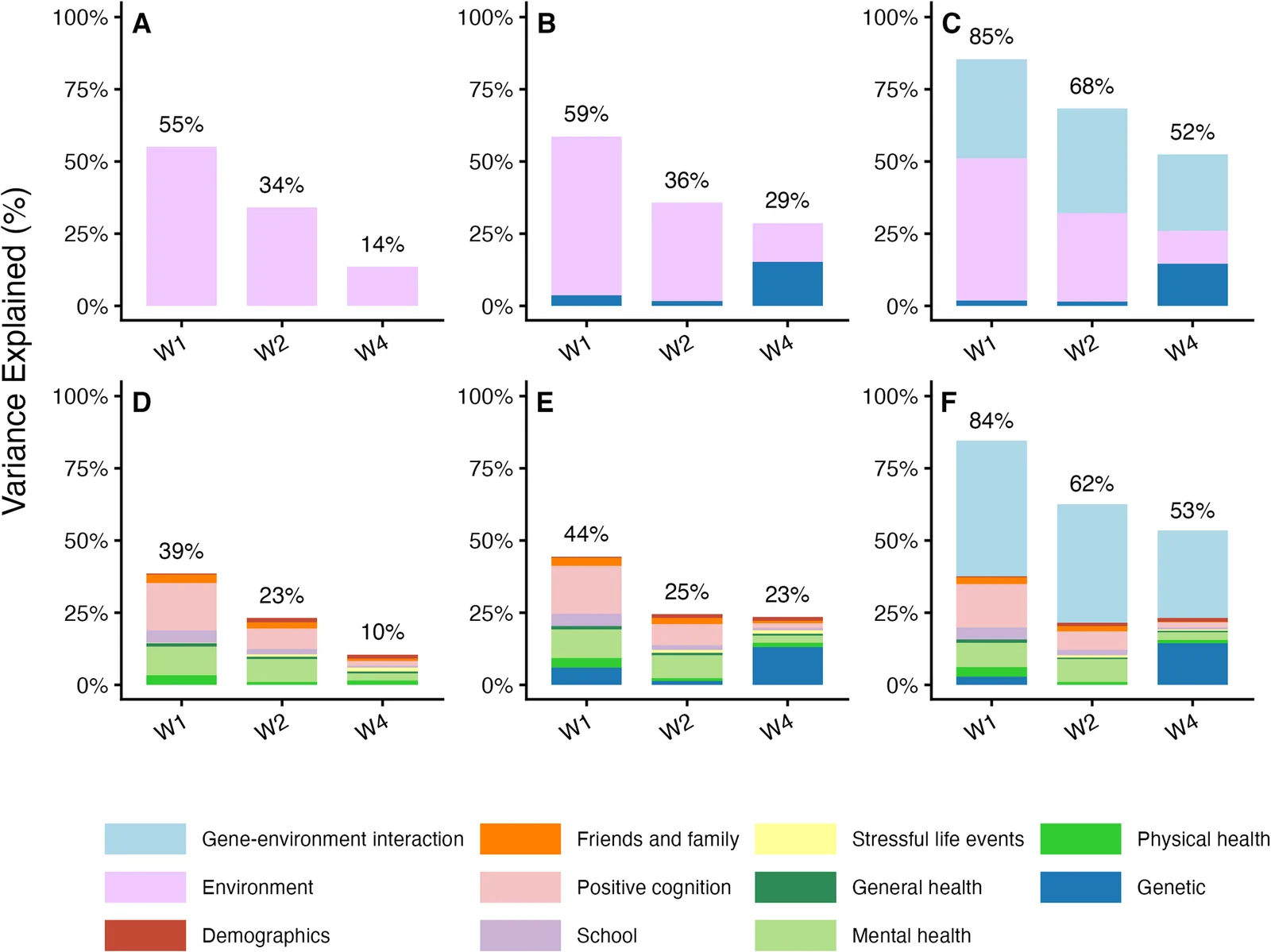
Proportion of variance explained (%) in depression symptoms scores in adolescence at Wave 1 and Wave 2 (W1, W2) and adulthood at Wave 4 (W4). *Panel A* shows the variance explained by 80 environmental factors modelled as a single random effect. *Panel B* shows the joint contribution of 80 environmental factors and genetic data, included simultaneously as two separate random effects in a single model. *Panel C* shows the joint contribution of 80 environmental factors and genetic factors, with an additional gene-environment interaction component (Box 1) included as a third random effect in the same model. *Panel D* shows the contribution of each environmental domain, modelled as separate random effects. *Panel E* shows the contribution of each environmental domain and genetic domain modelled separately. *Panel F* shows the contribution of each domain modelled as separate random effects, whilst including interaction effects between genetic and environmental domains

Environmental factors explained 55.06% (Standard Error [SE] = 2.20%) of the variance in adolescent depression symptoms at Wave 1, 34.07% (SE = 2.22%) one year later at Wave 2, and 13.57% (SE = 1.60%) in adulthood at Wave 4 (Fig. 2, **Panel A**). Genetic influences individually explained 26.07% (SE = 9.42%) at Wave 1, 16.24% (SE = 9.46%; *p* = 0.09) at Wave 2, and 24.96% (SE = 9.54%) at Wave 4 in adulthood.

When jointly estimating environmental and genetic influences, the environment mostly explained variation in depression symptoms. Genetic influences were non-significant and attenuated to 3.58% (SE = 4.26%; *p* = 0.40) at Wave 1, 1.72% (SE = 6.21%; *p* = 0.78) at Wave 2, and 15.20% (SE = 8.28%; *p* = 0.07) at Wave 4 (Fig. 2, **Panel B)**. The addition of the genetic variance component did not significantly improve model fit (Table 1). The genetic and environmental components were not correlated (W1 *r*=-0.36, *p* = 0.15; W2 *r*=-0.66, *p* = 0.61; W4 *r*=-0.07, *p* = 0.70) and modelling this covariance did not improve model fit (Table 1). However, gene-environment interaction components (Box 1) improved model fit and explained a further 34.33% (SE = 2.97%) of the variance at Wave 1, 36.33% (SE = 3.42%) at Wave 2, and 26.51% (SE = 3.81%) at Wave 4 (Fig. 2, **Panel C**). Accounting for gene-environment correlation did not change the contribution of these interaction components (W1 = 35.89%, W2 = 38.87%, and W4 = 26.76%), nor improve model fit.

We conducted two sets of sensitivity analyses for the gene-environment interaction model. First, we calculated the heritability of all environmental factors and removed the top ten most heritable from the environmental component. Second, we removed all health-related variables from the environmental component to assess if (general, mental, or physical) health factors were driving the variance explained. We found no substantial difference in the variance explained by the genetic, environmental, and interaction components for both sets of sensitivity analyses (**Supplement 2 A**).

### Domain-specific influences on depression symptoms

Different domains were associated with adolescent depression symptoms compared to adulthood (Fig. 2, **Panel D & E**). In adolescence, positive cognition (W1 16.46% and W2 7.18%) and mental health (W1 9.99% and W2 7.97%) domains contributed the most to depression symptoms (**Supplement 2B**). Followed by the school domain concurrently at Wave 1 (4.33%) and the friends and family domain one year later at Wave 2 (2.16%). Estimates should be interpreted as components of total phenotypic variance, i.e.,16.46% of the total variance in adolescent depression symptoms can be attributed to aligned similarity in positive cognition. Positive cognition items include if the participants like themselves, feel socially accepted, and feel loved and wanted. Mental health domain items are distinct but related to depression, such as fatigue, sleep problems, trouble relaxing, and fearfulness. In adulthood, all environmental domains contributed relatively equally small amounts to depression symptoms, including demographics (1.35%), positive cognition (1.60%), stressful life events (1.19%), mental health (2.45%) and physical health (1.58%). The genetic domain contributed substantially more to Wave 4 adulthood depression symptoms (13.05%), but this was non-significant (*p* = 0.12). The total variance explained did not decrease when we dropped the mental health domain from the model, but the contribution of the physical health domain increased across all Waves (W1 10.11%; W2 5.05%; W4 2.87%; **Supplement 2B.1**).

We initially allowed all domains to covary, which reduced individual contributions of each domain and instead suggested that this was attributable to covariance between domains (W1 20.48%, W2 19.38%, W4 15.45%). However, covariance model estimation was unstable (computing 36 additional parameters per outcome), and results are provided in **Supplement 2B**. Gene-by-environment domain interactions contributed substantially to the variance in depression symptoms across all waves (Fig. 2, **Panel F**) and improved model fit (Table 1). In adolescence, gene-by-environment domain interactions with mental health (W1 10.31% and W2 5.87%), positive cognition (W1 9.80% and W2 7.20%), physical health (W1 9.69% and W2 12.87%), friends and family (W1 8.32% and W2 6.08%), and school factors (W1 8.87% and W2 4.74%) contributed to depression symptoms (**Supplement 2B**). Whereas, only interactions with domains of friends and family (4.73%), mental health (11.16%), and stressful life events (7.05%) significantly contributed to depression symptoms in adulthood at Wave 4.

**Table 1:** Model fit comparisons using likelihood ratio tests

		Total variance explained	LKH	*p*	Improves fit?
**Total environment**					
W1	E only	55.06%	-1012.223	-	-
	G and E	58.61%	-1011.846	0.385	No
	G and E, rGE	54.09%	-1010.351	0.084	No
	G and E, GxE (compared to G and E)	85.46%	-929.884	< 0.001	Yes
	G and E, GxE, rGE (compared to GxE)	84.32%	-929.293	0.277	No
W2	E only	34.07%	-1534.594	-	-
	G and E	35.77%	-1534.556	0.781	No
	G and E, rGE	29.08%	-1532.749	0.057	No
	G and E, GxE (compared to G and E)	68.36%	-1468.027	< 0.001	Yes
	G and E, GxE, rGE (compared to GxE)	65.48%	-1466.594	0.090	No
W4	E only	13.57%	-1837.609	-	-
	G and E	28.66%	-1835.845	0.060	No
	G and E, rGE	27.49%	-1835.766	0.692	No
	G and E, GxE (compared to G and E)	52.49%	-1811.754	< 0.001	Yes
	G and E, GxE, rGE (compared to GxE)	52.03%	-1811.726	0.811	No
**Domains**					
W1	Domains	44.41%	-801.235	-	-
	Domains, DxD (compared to Domains)	83.63%	-704.648	< 0.001	Yes
W2	Domains	24.53%	-1388.547	-	-
	Domains, DxD (compared to Domains)	61.83%	-1316.014	< 0.001	Yes
W4	Domains	23.50%	-1774.722	-	-
	Domains, DxD (compared to Domains)	52.62%	-1743.418	< 0.001	Yes
**Systems**					
W1	Systems	53.04%	-912.231	-	-
	Systems, rS	52.59%	-888.054	< 0.001	Yes
	Systems, SxS (compared to Systems)	83.56%	-844.607	< 0.001	Yes
	Systems, rS & SxS (compared to SxS)	80.46%	-831.200	< 0.001	Yes
W2	Systems	27.13%	-1460.011	-	-
	Systems, rS	26.33%	-1445.933	< 0.001	Yes
	Systems, SxS (compared to Systems)	64.36%	-1407.309	< 0.001	Yes
	Systems, rS & SxS (compared to SxS)	54.72%	-1395.278	< 0.001	Yes
W4	Systems	29.21%	-1813.149	-	-
	Systems, rS	29.73%	-1794.830	< 0.001	Yes
	Systems, SxS (compared to Systems)	48.14%	-1798.104	< 0.001	Yes
	Systems, rS & SxS (compared to SxS)	44.97%	-1794.244	0.0521	No

Note. W1 = reduced CES-D score at Wave 1; W2 = reduced CES-D score at Wave 2; W4 = reduced CES-D score at Wave 4; LKH = log-likelihood; p = p value of significance; G = genetic influence; E = environmental influence; rGE = gene-environment correlation; GxE = gene-environment interaction; rD = correlation between domains; DxD = domain by domain interaction; rS = correlation between systems; SxS = system by system interaction. Additional parameters from baseline models: DxD = 8; rS = 2; SxS = 3; rS & SxS = 3.

### To what extent can large-scale data explain depression symptoms?

Systems explained 53.04% of the variance in adolescent depression symptoms (W1), 27.13% one year later (W2), and 29.21% in adulthood depression symptoms (W4). Figure 4 shows the relative percentage of the variance explained by each system for the three depression scores. At Wave 1, only psychological (42.72%) and social systems (5.38%) significantly contributed to depression symptoms. At Wave 2, psychological (22.63%), social (6.74%), and built (0.65%) systems significantly contributed. Whereas, at Wave 4, genetic (16.64%), psychological (7.93%), and social (4.00%) systems significantly contributed to depression symptoms. Across all Waves, the natural system did not contribute to depression symptoms (**Supplement 2 C**).

Accounting for genetic-psychological and psychological-social covariance components (W1 *r* = 0.45; W2 *r* = 0.40; W4 *r* = 0.36) improved model fit (Table 1) but did not change the total variance explained from the systems-only model (**Supplement 2 C**). However, gene-by-system interactions with psychological, social, and built systems increased the variance explained by 30.52% at W1, 37.23% at W2, and 18.93% at W4, and improved model fit. Genetic-psychological system interactions (W1 26.19%; W2 23.48%; W4 12.06%), and genetic-social system interactions (W1 9.91%; W2 10.93%; W4 9.50%) contributed most to depression symptoms across all Waves, even when controlling for genetic-psychological, genetic-social, and psychological-social correlations (**Supplement 2 C**). When we removed the psychological system, the variance explained remained consistent but was instead attributed to social systems (**Supplement 2 C.1**).

**Fig. 3:**
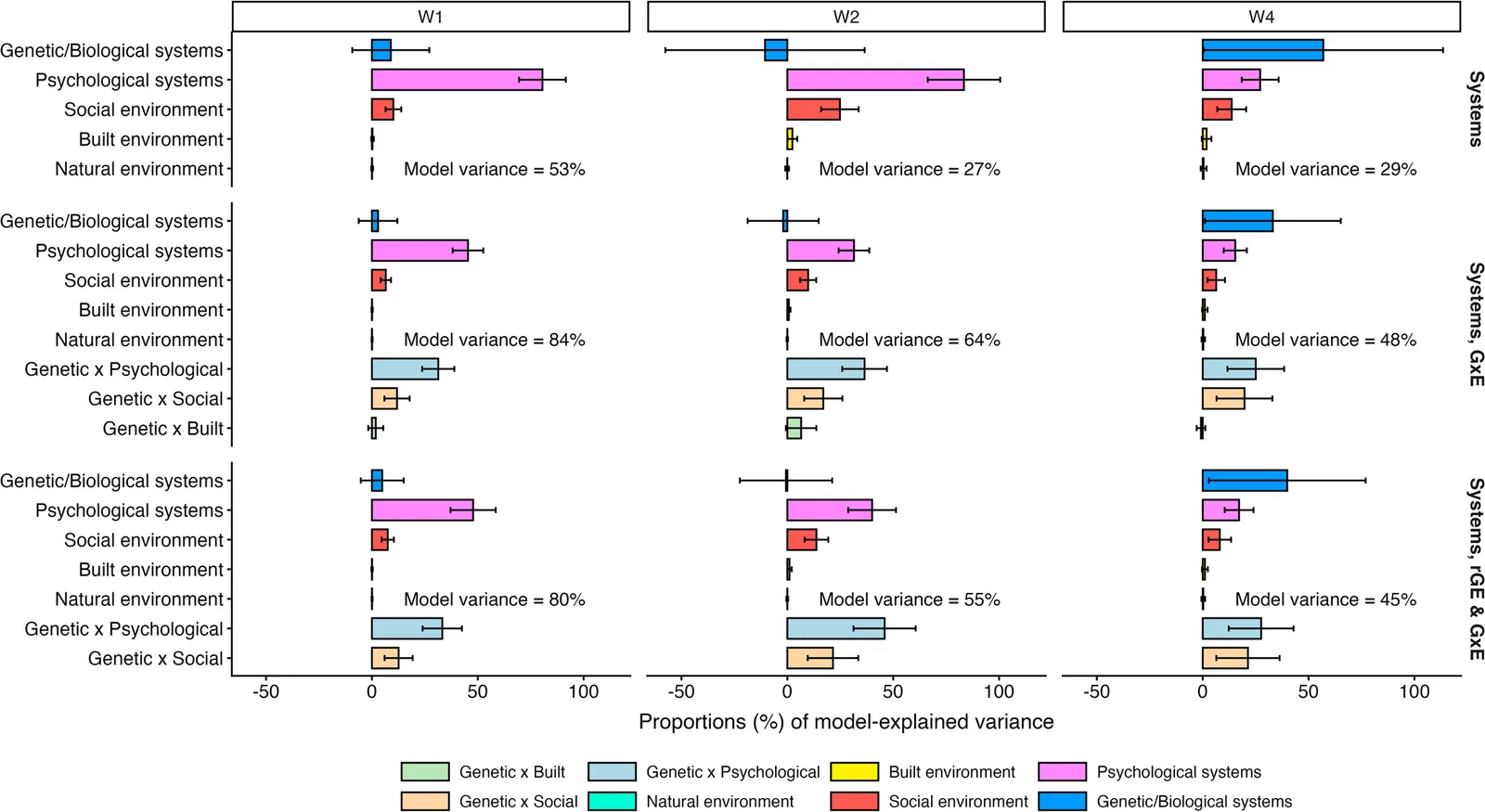
Relative percentage of variance explained in depression symptoms scores concurrently during adolescence (W1), one year later (W2), and adulthood (W4). The total variance explained is shown for each model. The contribution of each system is calculated as the standardised variance explained divided by the total variance explained in the model, multiplied by 100. This represents the proportion of variance each system accounts for relative to the total variance explained within that model. Each system is listed on the left. The top panel (“Systems” model) includes each system as an independent random effect. The middle panel (“Systems, GxE” model) includes gene-environment interaction components with psychological, social, and built environments. The bottom panel (“Systems, rGE & GxE” model) includes significant gene-psychological and gene-social interactions whilst additionally accounting for genetic-psychological, genetic-social, and psychological-social covariances. Out-of-bounds components are ones in which unstable model estimation resulted in negative variances, missing standard errors, or computed correlations that were +/-1. Variance components can have small negative values, which are likely due to sample variability (if the random effects are weak), which can push an estimate below zero.

## Discussion

We provide a holistic insight into the plethora of depression risk factors through jointly modelling people’s experiences, characteristics, and surroundings. We first assessed how individual-level risk factors contributed to depression symptoms. We found that for adolescent depression, positive cognition (feeling accepted and loved) outweighed the influence of other factor domains. Whereas all domains contributed equally to adulthood symptoms over a decade later. Second, we integrated multiple data sources to assess contributions of genetic, psychological, social, built, and natural systems. Built (political, healthcare, poverty, and crime) and natural systems (population density, urbanicity, and weather) explained little variation in depression symptoms after accounting for genetic, psychological, and social systems. Our results point towards a complex interplay between genetic and psychological processes. We demonstrate how to integrate broad yet detailed cohort data to understand depression risk, and emphasise the value of a nuanced, multisystemic perspective.

Our findings highlight the role of positive cognition in young people’s depression symptoms, even when accounting for demographics, family relationships, school environments, mental health, physical health, general health, genetic liability, and stressful life events. The positive cognition domain included questions on feeling socially accepted, feeling loved and wanted, and liking yourself. This reflects the core notion of traditional cognitive theories [39] and current cognitive-behavioural interventions [40]. Maladaptive schemas around the self, world, and future can form cognitive vulnerabilities that increase depressive susceptibility. Depression has been consistently associated with the quality and perception of social support, contact, and acceptance from society, family, and peers [41]. Psychological factors were also most prominent when modelling different systems, which is consistent with other research using machine learning [42] and longitudinal approaches [15].

The emphasis on psychological factors should not be interpreted as evidence that built and natural systems are unimportant for the mental health of young people. Instead, their contribution may be more difficult to detect and underestimated due to the structure of this US-based data. For example, due to geocoded county-level aggregation, limited within-area variability, and residential mobility, which can attenuate more localised or time-varying influences of these environments. Moreover, the impact of the built environment through societal inequalities, such as poverty and access to healthcare, could be more integral later as adults cope with the stress of obtaining financial resources to have their needs met [8, 43].

We found a developmental pattern in domain associations with depression symptoms. Positive cognition contributed most in adolescence (both concurrently and one year later), whereas effects were evenly distributed across domains in early adulthood. These findings could suggest three things. First, adolescence is a period when identity formation, social comparison, and self-evaluation may be particularly central to mental health [44]. Second, shared-method variance, exposure-outcome proximity, and symptom-related reporting effects may influence these associations, as depressive symptoms can shape how individuals perceive and report their experiences [45]. Third, life-course changes will lead to the emergence of adult-specific exposures. For example, in the thirteen-year gap between Wave 1 and Wave 4, individuals may experience entering employment, financial responsibility, romantic relationships, independent living, and health-related responsibilities, all of which could play a role in depression symptoms in adulthood.

Using large-scale environmental data, we explained a higher proportion of variance in depression symptoms than previous estimates from cumulative score approaches [10, 46], relatedness-matrix approaches [33], and longitudinal studies using a reduced set of factors [47]. Furthermore, we found that genetic influence and psychological systems interact and jointly contribute to shaping depression symptoms, particularly across time. This intuitively captures whether genetic similarity can explain similarity in depression symptoms, particularly among people who also share similar environments. In other words, environmental similarity moderates the extent to which genetic similarity translates into phenotypic similarity across individuals. Genetic influence on depression could be reduced for individuals with high psychological functioning, or the impact of self-acceptance on depression could be stronger for those with high genetic liability. This supports multisystemic approaches, which suggest complex interactions between levels of biological, psychological, and social data [12] and addresses calls to integrate these systems [48]. People with high depression genetic risk under adverse social conditions have been shown to benefit more from nurturing social environments [49]. Creating accepting and supportive environments for young people [50, 51] could be particularly beneficial for those with elevated depression genetic risk [52].

This research has limitations. First, we do not capture time-dynamics in risk factors. A young person’s life and symptoms are not static, and our current risk factors measured at Wave 1 only include a single snapshot in time and precede the depression symptoms assessed at Wave 4 by a relatively long interval. Second, self-report environmental factors are subject to self-rater bias, which may inflate variance components from these domains compared to those based on objectively measured data. Third, several items (e.g. sexual assault or frequency of period pains) were only asked of females. Fourth, there is conceptual overlap between the psychological domain and depression symptoms, which can inflate concurrent estimates for this domain. Therefore, it is also possible that our findings reflect cyclic or bidirectional associations that were not appropriately captured here. Fifth, due to the large-scale longitudinal nature of our data, the environmental measures reflect young people’s experiences and contexts in the 1990s, which may not match those of today (e.g., due to the rise of technology). Sixth, it is unclear how our findings would differ for clinical populations and people of non-European-like ancestries. Genetic findings based on European ancestry samples are not generalisable to non-European ancestries due to differences in allele frequencies, linkage disequilibrium structure, and genetic architecture across populations. Genetic and environmental effects presented here will differ in other samples of different ages, ancestries, races, cultures, measures, and clinical sampling. We draw from evidence of risk factors primarily from the Global North, and research is needed across global contexts. Seventh, given that these models are data-driven and covariance-based, our findings do not establish causality or assess prediction. Eighth, each Wave of data collection in Add Health spans an age range of eight years, which may introduce bias; however, year of birth was included as a covariate in all analyses.

Our findings have implications for public health and future research. It is well known that increasing social support, connection, and acceptance is beneficial for mental health, particularly for vulnerable people [53, 54]. Our results align with this understanding and place the relative contribution of feeling supported within the context of a broad array of risk factors. Campaigns to increase social connection and community inclusion are on the rise, with affirming messaging across schools, workplaces, healthcare, and media, but wide-reaching initiatives are difficult to achieve [55]. Our findings provide additional support for ongoing social intervention efforts aimed at strengthening social connection and inclusion among young people [56]. However, to translate these findings to intervention, more work is required using causal designs. Future research can build on our findings by incorporating complex longitudinal structures within bigger data in diverse populations. Time-sensitive analyses would also provide nuance and insight into directionality, dynamics, and changes over time in the contributions of different domains of risk factors.

## Supplementary Information

Below is the link to the electronic supplementary material.


Supplementary Material 1


## Data Availability

This research uses data from Add Health. Access to the restricted-use Add Health data is available to approved researchers through an application process. More information about this process can be found here: https://addhealth.cpc.unc.edu/data/restricted-use-data-sets/. Our Add Health phenotypic data was applied for and approved under restricted-use application #27111802. The Add Health genetic data was applied for and approved under application #38868 using the National Institutes of Health (NIH) National Center for Biotechnology Information (NCBI)’s database of Genotypes and Phenotypes (dbGaP). Use of the Add Health data at Purdue University was approved by the Purdue Institutional Review Board (IRB) under application IRB-2023-1819. All analyses were pre-registered on the Open Science Framework (https://osf.io/akzhd/overview), and code is available on GitHub (https://github.com/knthompson26/findmedep).
