# Supplementary material for: Illuminating the complex interplay of risk factors for depression symptoms within a large-scale US longitudinal cohort
Source: Soc Psychiatry Psychiatr Epidemiol. 2026 May 5;61(8):1451–62. doi: 10.1007/s00127-026-03098-0 (PMC13522009; doi:10.1007/s00127-026-03098-0)
Supplement: Supplementary file 1 [file 127_2026_3098_MOESM1_ESM.docx]

**Supplementary material**

**Illuminating the complex interplay of risk factors for depression within a large-scale US longitudinal cohort**

[Supplement 1. Methods	2](#_Toc223961334)

[*1A. Domain-level variables used for research question 1*	2](#_Toc223961335)

[*1B. System-level variables used for research question 2*	6](#_Toc223961336)

[*1C. Missingness for predictors*	16](#_Toc223961337)

[*1D. Statistical analyses*	17](#_Toc223961338)

[How do prominent risk factors for depression contribute to symptom occurrence?	19](#_Toc223961339)

[To what extent can large-scale environmental data explain depression symptoms?	20](#_Toc223961340)

[Supplement 2. Results	20](#_Toc223961341)

[*Descriptive statistics*	20](#_Toc223961342)

[*2A. Broad environmental and genetic influences on depression symptoms*	22](#_Toc223961343)

[2A.1 Sensitivity analysis: Top 10 heritable factors removed	23](#_Toc223961344)

[2A.2 Sensitivity analysis: Health factors removed	25](#_Toc223961345)

[*2B. Domain specific influences on depression symptoms*	25](#_Toc223961346)

[2B.1 Sensitivity analysis: Mental health domain removed	29](#_Toc223961347)

[*2C. System-specific influences on depression symptoms*	30](#_Toc223961348)

[2C.1 Sensitivity analysis: Reduced systems models	31](#_Toc223961349)

# Supplement 1. Methods

Add Health participants were recruited across 80 high schools and completed five waves of data collection across 20 years. Wave 1 was collected in 1994-1995 (individuals aged 12-20; N=20,745), Wave 2 was conducted one year later (N=14,738), Wave 3 from 2001 to 2002 (19-26; N=15,197), Wave 4 in 2008 (24-32; N=15,701), and Wave 5 from 2016-2018 (34-42; N=~12,000). Ethical review was performed by the Purdue University Institutional Review Board (IRB-2023-1819) and data securely stored on the encrypted Purdue Rossmann Cluster.  Informed consent was obtained from all participants. Wave 1 contained 19 items from the full 20 scale which omitted items of “My sleep was restless” and “I had crying spells” and included an additional item of “You felt life was not worth living”. Wave 2 and 4 contained 10 of the core CES-D items which focused on mood symptoms.  Responses for each item were 0 (never or rarely), 1, (sometimes), 2 (a lot of the time), and 3 (most or all the time). Participants could also select “I refuse” and “I don’t know” or skip the item. We removed individuals who had more than one item missing. We summed all valid responses to each item to create four severity scores representing depression symptoms: W1A with 19 items at Wave I; W1B with 10 items at Wave 1; W2 with the same 10 items at Wave 2; and W4 with the same 10 items at Wave 4. Only four people were missing more than one item at Wave 1, 23 at Wave 2, and 4 at Wave 4.

## *1A. Domain-level variables used for research question 1*

| **Table 1.** Domain variables (80 variables + 1 that represents genetic data) | | | |
| --- | --- | --- | --- |
| **Domain** | **Variable name** | **Description** | **Range** |
| Covariate | BIO_SEX | biological sex (binary: male=1/female=2) | binary: male=1, female=2 |
|  | Ancestry PCs 1-20 | PC1-PC20 | Continuous: -0.9-0.86 |
|  | H1GI1Y | Year of birth | Numeric: 1974-1983 |
| Demographics | PA55 | total family income before taxes - all incomes and sources ($1000) | Numeric: distribution spikes on tens; 0-999 |
|  | PA56 | do you have enough money to pay your bills | Binary: Yes=1, no=0 |
|  | PA59 | how easy/hard is it to get medical care for your family | Categorical ordered: very easy,somewhat easy, somewhat hard, very hard |
|  | H1NB5 | do you usually feel safe in your neighbourhood | binary: Yes=1, no=0 |
|  | H1NB6 | how happy are you with living in your neighbourhood | Categorical ordered: not at all, very little, somewhat, quite a bit, very much |
|  | H1GH27I | reason for not seeking healthcare - couldn't pay, No to H1GH26 | Binary: Yes to couldn't pay, No to couldn't pay |
| General health | H1GH1 | general health | Categorical ordered: excellent, very good, good, fair, poor |
|  | H1GH26 | thought needed medical care but didn't seek it - past year | Binary: Yes=1, No=0 |
|  | H1GH48 | health or emotional problem caused day off school | Categorical ordered: Never, a few times, once a week, almost every day, every day |
|  | H1GH49 | health or emotional problem caused miss recreational activity | Categorical ordered: Never, a few times, once a week, almost every day, every day |
| Mental health | H1GH8 | frequency of fatigue - past 12 months | Categorical ordered: Never, just a few times, once a week, almost every day, every day |
|  | H1GH18 | frequency of sleep problems - past 12 months | Categorical ordered: Never, just a few times, once a week, almost every day, every day |
|  | H1GH19 | frequency of trouble relaxing - past 12 months | Categorical ordered:Never, just a few times, once a week, almost every day, every day |
|  | H1GH22 | frequency of fearfulness - past 12 months | Categorical ordered: Never, just a few times, once a week, almost every day, every day |
|  | H1HS3 | psychological counselling in past year | Binary: yes=1, no=0 |
| Physical health | H1PL1 | permanent physical condition | Binary: Yes=1, no=0 |
|  | H1GH2 | frequency of headache - past 12 months | Never, just a few times, once a week, almost every day, every day |
|  | H1GH3 | frequency of feeling hot - past 12 months | Categorical ordered:Never, just a few times, once a week, almost every day, every day |
|  | H1GH4 | frequency of stomach ache - past 12 months | Categorical ordered:Never, just a few times, once a week, almost every day, every day |
|  | H1GH5 | frequency of cold sweats - past 12 months | Categorical ordered:Never, just a few times, once a week, almost every day, every day |
|  | H1GH6 | frequency of feeling weak - past 12 months | Categorical ordered:Never, just a few times, once a week, almost every day, every day |
|  | H1GH7 | frequency of sore throat - past 12 months | Categorical ordered:Never, just a few times, once a week, almost every day, every day |
|  | H1GH9 | frequency of painful urination - past 12 months | Categorical ordered:Never, just a few times, once a week, almost every day, every day |
|  | H1GH10 | frequency of feeling really sick - past 12 months | Categorical ordered:Never, just a few times, once a week, almost every day, every day |
|  | H1GH11 | frequency of waking up tired - past 12 months | Categorical ordered:Never, just a few times, once a week, almost every day, every day |
|  | H1GH12 | frequency of skin problems - past 12 months | Categorical ordered:Never, just a few times, once a week, almost every day, every day |
|  | H1GH13 | frequency of dizziness - past 12 months | Categorical ordered: Never, just a few times, once a week, almost every day, every day |
|  | H1GH14 | frequency of chest pains - past 12 months | Categorical ordered: Never, just a few times, once a week, almost every day, every day |
|  | H1GH15 | frequency of aches and pains - past 12 months | Categorical ordered: Never, just a few times, once a week, almost every day, every day |
|  | H1GH16 | frequency of period pains - past 12 months | Categorical ordered: Never, just a few times, once a week, almost every day, every day |
|  | H1GH17 | frequency of poor appetite - past 12 months | Categorical ordered: Never, just a few times, once a week, almost every day, every day |
|  | H1GH28 | how do you think of yourself in weight | Categorical ordered:1=very underweight, slightly underweight, about right, slightly overweight, 5=very overweight |
|  | H1GH29 | trying to change weight | Categorical ordered: lose weight, gain weight, stay the same weight, not trying to do anything about weight |
| Difficulties at school | H1ED15 | trouble getting along with teachers - past year | Categorical ordered: Never, a few times, once a week, almost every day, every day |
|  | H1ED16 | trouble paying attention in school - past year | Categorical ordered: Never, a few times, once a week, almost every day, every day |
|  | H1ED17 | trouble getting homework done - past year | Categorical ordered: Never, a few times, once a week, almost every day, every day |
|  | H1ED18 | trouble getting along with other students - past year | Categorical ordered: Never, a few times, once a week, almost every day, every day |
|  | H1ED19 | feel close to people at school - past year | Categorical ordered: Never, a few times, once a week, almost every day, every day |
|  | H1ED20 | feel like you are part of your school - past year | Categorical ordered: Never, a few times, once a week, almost every day, every day |
|  | H1ED21 | students at school are prejudiced - past year | Categorical ordered: Never, a few times, once a week, almost every day, every day |
|  | H1ED22 | happy to be at your school - past year | Categorical ordered: Never, a few times, once a week, almost every day, every day |
|  | H1ED23 | teachers treat students fairly - past year | Categorical ordered: Never, a few times, once a week, almost every day, every day |
|  | H1ED24 | feel safe in your school - past year | Categorical ordered:Never, a few times, once a week, almost every day, every day |
| Relationships with friends and family | H1DA7 | frequency hanging out with friends | Categorical ordered:not at all, 1 or 2 times, 3 or 4 times, 5 or more times |
|  | H1WP9 | how close do you feel to your mother | Categorical ordered:not at all, very little, somewhat, quite a bit, very much |
|  | H1WP10 | how much do you think she cares about you | Categorical ordered: not at all, very little, somewhat, quite a bit, very much |
|  | H1WP13 | how close to you feel to your father - lots missing. | Categorical ordered: not at all, very little, somewhat, quite a bit, very much |
|  | H1WP14 | how much to you think he cares about you - lots missing. | Categorical ordered: not at all, very little, somewhat, quite a bit, very much |
|  | H1PF1 | your mother is warm and loving to you | Categorical ordered:strongly agree, agree, neither agree or disagree, disagree, strongly disagree |
|  | H1PF4 | satisfied with the way you and your mother communicate | Categorical ordered: strongly agree, agree, neither agree or disagree, disagree, strongly disagree |
|  | H1PF5 | satisfied with your relationships with your mother | Categorical ordered:strongly agree, agree, neither agree or disagree, disagree, strongly disagree |
|  | H1PF24 | satisfied with the way you and your father communicate - lots missing. | Categorical ordered: strongly agree, agree, neither agree or disagree, disagree, strongly disagree |
|  | H1PF25 | satisfied with your relationship with your father - lots missing. | Categorical ordered: strongly agree, agree, neither agree or disagree, disagree, strongly disagree |
|  | H1PR1 | feel that adults care about you | Categorical ordered: not at all, very little, somewhat, quite a bit, very much, does not apply |
|  | H1PR2 | feel your teachers care about you | Categorical ordered:not at all, very little, somewhat, quite a bit, very much, does not apply |
|  | H1PR3 | feel your parents care about you | Categorical ordered: not at all, very little, somewhat, quite a bit, very much, does not apply |
|  | H1PR4 | feel your friends care about you | Categorical ordered: not at all, very little, somewhat, quite a bit, very much, does not apply |
|  | H1PR5 | feel people in your family understand you | Categorical ordered: not at all, very little, somewhat, quite a bit, very much, does not apply |
|  | H1PR6 | feel you want to leave home | Categorical ordered: not at all, very little, somewhat, quite a bit, very much, does not apply |
|  | H1PR7 | feel that you and your family have fun together | Categorical ordered: not at all, very little, somewhat, quite a bit, very much, does not apply |
|  | H1PR8 | feel that your family pays attention to you | Categorical ordered: not at all, very little, somewhat, quite a bit, very much, does not apply |
| Positive cognition and social acceptance | H1PF33 | you like yourself | Categorical ordered: strongly agree, agree, neither agree or disagree, disagree, strongly disagree |
|  | H1PF34 | you feel like you are doing everything just about right | Categorical ordered: strongly agree, agree, neither agree or disagree, disagree, strongly disagree |
|  | H1PF35 | you feel socially accepted | Categorical ordered: strongly agree, agree, neither agree or disagree, disagree, strongly disagree |
|  | H1PF36 | you feel loved and wanted | Categorical ordered: strongly agree, agree, neither agree or disagree, disagree, strongly disagree) |
| Stressful life events / traumatic experiences | H1CO10 | ever been raped - female only -lifetime | Categorical ordered: 0=not raped and female, 1= raped and female, 2=Male and not possible to be raped |
|  | H1NR3 | ever given someone sex in exchange for drugs or money -lifetime | Binary: Yes=1, No=0 |
|  | H1FV1 | saw someone shoot or stab another person - past year | Categorical ordered: never, once, more than once |
|  | H1FV2 | someone pulled a knife or gun on you - past year | Categorical ordered: never, once, more than once |
|  | H1FV3 | someone shot you - past year | Categorical ordered: never, once, more than once |
|  | H1FV4 | someone cut or stabbed you - past year | Categorical ordered: never, once, more than once |
|  | H1FV5 | you got into a physical fight - past year | Categorical ordered: never, once, more than once |
|  | H1FV6 | you were jumped - past year | Categorical ordered: never, once, more than once |
|  | H1FP21_1 | how did pregnancy 1 end - lifetime | Categorical ordered: abortion, live birth, hasn't ended, still birth/miscarriage |
|  | H1FP21_2 | how did pregnancy 2 end -lifetime | Categorical ordered: abortion, live birth, hasn't ended, still birth/miscarriage |
|  | H1FP21_3 | how did pregnancy 3 end - lifetime | Categorical ordered: abortion, live birth, hasn't ended, still birth/miscarriage |
|  | H1SU4 | have any of your friends tried to kill themselves - past 12 months | Binary: Yes=1, No=0 |
|  | H1SU5 | Have any of your friends have succeeded in committing suicide in the past 12 months? | Binary: Yes=1, No=0 |
|  | H1SU6 | have any of your family members tried to kill themselves - past 12 months | Binary: Yes=1, No=0 |
|  | H1SU7 | Have any of your family members succeeded in committing suicide in the past 12 months | Binary: Yes=1, No=0 |

## *1B. System-level variables used for research question 2*

The purpose of the REML-based approach to environmental data is to enable research to include many variables into one model. As an exemplification for how to do this - we include four large sources of data from Add Health and structure them according to a systems perspective in our model. The four data sources include: In-home questionnaire data, contextual data, neighbourhood data, political data, and county health data. These data were then structured within the model according to a systems theory perspective. In total there were 161 environmental variables + 1 variable representing genetic data + 3 covariates.

| **Table 2. System-level variables (161 +1 that represents genetic data)** | | | | |
| --- | --- | --- | --- | --- |
| **Domain** | **Variable** | **Notes** | **Range** | **Included in RQ1** |
| **Covariate** | | | | |
|  | BIO_SEX | biological sex | Binary: male=1, female=2 | yes |
|  | Ancestry PCs 1-20 | PC1-PC20 | Numeric: -0.9-0.86 | yes |
|  | H1GI1Y | Year of birth | Numeric: 1974-1983 | yes |
| **Biological systems** | | | | |
|  | Genetic influence | SNP data | Numeric | yes |
| **Psychological systems** | | | | |
| General, mental, and physical health | H1GH1 | general health | Categorical ordered: excellent, very good, good, fair, poor | yes |
|  | H1GH26 | thought needed medical care but didn't seek it - past year | Binary: Yes=1, No=0 | yes |
|  | H1GH48 | health or emotional problem caused day off school | Categorical frequency: Never, a few times, once a week, almost every day, every day | yes |
|  | H1GH49 | health or emotional problem caused miss recreational activity | Categorical frequency: Never, a few times, once a week, almost every day, every day | yes |
|  | H1GH8 | frequency of fatigue - past 12 months | Categorical ordered: Never, just a few times, once a week, almost every day, every day | yes |
|  | H1GH18 | frequency of sleep problems - past 12 months | Categorical ordered: Never, just a few times, once a week, almost every day, every day | yes |
|  | H1GH19 | frequency of trouble relaxing - past 12 months | Categorical ordered: Never, just a few times, once a week, almost every day, every day | yes |
|  | H1GH22 | frequency of fearfulness - past 12 months | Categorical ordered: Never, just a few times, once a week, almost every day, every day | yes |
|  | H1HS3 | psychological counselling in past year | Binary: yes=1, no=0 | yes |
|  | H1PL1 | permanent physical condition | Binary: Yes=1, no=0 | yes |
|  | H1GH2 | frequency of headache - past 12 months | Categorical ordered: Never, just a few times, once a week, almost every day, every day | yes |
|  | H1GH3 | frequency of feeling hot - past 12 months | Categorical ordered: Never, just a few times, once a week, almost every day, every day | yes |
|  | H1GH4 | frequency of stomach ache - past 12 months | Categorical ordered: Never, just a few times, once a week, almost every day, every day | yes |
|  | H1GH5 | frequency of cold sweats - past 12 months | Categorical ordered: Never, just a few times, once a week, almost every day, every day | yes |
|  | H1GH6 | frequency of feeling weak - past 12 months | Categorical ordered: Never, just a few times, once a week, almost every day, every day | yes |
|  | H1GH7 | frequency of sore throat - past 12 months | Categorical ordered: Never, just a few times, once a week, almost every day, every day | yes |
|  | H1GH9 | frequency of painful urination - past 12 months | Categorical ordered: Never, just a few times, once a week, almost every day, every day | yes |
|  | H1GH10 | frequency of feeling really sick - past 12 months | Categorical ordered: Never, just a few times, once a week, almost every day, every day | yes |
|  | H1GH11 | frequency of waking up tired - past 12 months | Categorical ordered: Never, just a few times, once a week, almost every day, every day | yes |
|  | H1GH12 | frequency of skin problems - past 12 months | Categorical ordered: Never, just a few times, once a week, almost every day, every day | yes |
|  | H1GH13 | frequency of dizziness - past 12 months | Categorical ordered: Never, just a few times, once a week, almost every day, every day | yes |
|  | H1GH14 | frequency of chest pains - past 12 months | Categorical ordered: Never, just a few times, once a week, almost every day, every day | yes |
|  | H1GH15 | frequency of aches and pains - past 12 months | Categorical ordered: Never, just a few times, once a week, almost every day, every day | yes |
|  | H1GH16 | frequency of period pains - past 12 months | Categorical ordered: Never, just a few times, once a week, almost every day, every day | yes |
|  | H1GH17 | frequency of poor appetite - past 12 months | Categorical ordered: Never, just a few times, once a week, almost every day, every day | yes |
|  | H1GH28 | how do you think of yourself in weight | Categorical ordered: 1=very underweight, slightly underweight, about right, slightly overweight, 5=very overweight | yes |
|  | H1GH29 | trying to change weight | Categorical: lose weight, gain weight, stay the same weight, not trying to do anything about weight | yes |
| Positive cognition | H1PF33 | you like yourself | Categorical: strongly agree, agree, neither agree or disagree, disagree, strongly disagree | yes |
|  | H1PF34 | you feel like you are doing everything just about right | Categorical: strongly agree, agree, neither agree or disagree, disagree, strongly disagree | yes |
|  | H1PF35 | you feel socially accepted | Categorical: strongly agree, agree, neither agree or disagree, disagree, strongly disagree | yes |
|  | H1PF36 | you feel loved and wanted | Categorical: strongly agree, agree, neither agree or disagree, disagree, strongly disagree | yes |
| Additional personality items measured in Wave I identified by Young and Beaujean (2011)  DOI:10.3389/fpsyg.2011.00158 | H1PF30 | You have a lot of good qualities | Categorical: strongly agree, agree, neither agree or disagree, disagree, strongly disagree | no |
|  | H1PF32 | You have a lot to be proud of | Categorical: strongly agree, agree, neither agree or disagree, disagree, strongly disagree | no |
|  | H1PF18 | When you have a problem to solve, one of the first things you do is get as many facts about the problem as possible | Categorical: strongly agree, agree, neither agree or disagree, disagree, strongly disagree | no |
|  | H1PF19 | When you are attempting to find a solution to a problem, you usually try to think of as many different ways to approach the problem as possible | Categorical: strongly agree, agree, neither agree or disagree, disagree, strongly disagree | no |
|  | H1PF20 | When making decisions, you generally use a systematic method for judging and comparing alternatives | Categorical: strongly agree, agree, neither agree or disagree, disagree, strongly disagree | no |
|  | H1PF21 | After carrying out a solution to a problem, you usually try to analyze what went right and what went wrong | Categorical: strongly agree, agree, neither agree or disagree, disagree, strongly disagree | no |
| **Social environment** | | | | |
| Neighbourhood | H1NB5 | do you usually feel safe in your neighbourhood | Binary: Yes=1, no=0 | yes |
|  | H1NB6 | how happy are you with living in your neighbourhood | Categorical ordered: not at all, very little, somewhat, quite a bit, very much | yes |
| School | H1ED15 | trouble getting along with teachers - past year | Categorical frequency: Never, a few times, once a week, almost every day, every day | yes |
|  | H1ED16 | trouble paying attention in school - past year | Categorical frequency: Never, a few times, once a week, almost every day, every day | yes |
|  | H1ED17 | trouble getting homework done - past year | Categorical frequency: Never, a few times, once a week, almost every day, every day | yes |
|  | H1ED18 | trouble getting along with other students - past year | Categorical frequency: Never, a few times, once a week, almost every day, every day | yes |
|  | H1ED19 | feel close to people at school - past year | Categorical frequency: Never, a few times, once a week, almost every day, every day | yes |
|  | H1ED20 | feel like you are part of your school - past year | Categorical frequency: Never, a few times, once a week, almost every day, every day | yes |
|  | H1ED21 | students at school are prejudiced - past year | Categorical frequency: Never, a few times, once a week, almost every day, every day | yes |
|  | H1ED22 | happy to be at your school - past year | Categorical frequency: Never, a few times, once a week, almost every day, every day | yes |
|  | H1ED23 | teachers treat students fairly - past year | Categorical frequency: Never, a few times, once a week, almost every day, every day | yes |
|  | H1ED24 | feel safe in your school - past year | Categorical frequency: Never, a few times, once a week, almost every day, every day | yes |
|  | H1PR2 | feel your teachers care about you | Categorical ordered: not at all, very little, somewhat, quite a bit, very much, does not apply | yes |
| Friends | H1DA7 | frequency hanging out with friends | Categorical frequency: not at all, 1 or 2 times, 3 or 4 times, 5 or more times | yes |
|  | H1PR4 | How much do you feel that your friends care about you? | Categorical ordered: not at all, very little, somewhat, quite a bit, very much, does not apply | yes |
| Family | H1WP9 | how close do you feel to your mother | Categorical frequency: not at all, very little, somewhat, quite a bit, very much | yes |
|  | H1WP10 | how much do you think she cares about you | Categorical frequency: not at all, very little, somewhat, quite a bit, very much | yes |
|  | H1WP13 | how close do you feel to your father - lots missing. | Categorical frequency: not at all, very little, somewhat, quite a bit, very much | yes |
|  | H1WP14 | how much to you think he cares about you - lots missing. | Categorical frequency: not at all, very little, somewhat, quite a bit, very much | yes |
|  | H1PF1 | your mother is warm and loving to you | Categorical frequency: strongly agree, agree, neither agree or disagree, disagree, strongly disagree | yes |
|  | H1PF4 | satisfied with the way you and your mother communicate | Categorical frequency: strongly agree, agree, neither agree or disagree, disagree, strongly disagree | yes |
|  | H1PF5 | satisfied with your relationships with your mother | Categorical frequency: strongly agree, agree, neither agree or disagree, disagree, strongly disagree | yes |
|  | H1PF24 | satisfied with the way you and your father communicate - lots missing. | Categorical frequency: strongly agree, agree, neither agree or disagree, disagree, strongly disagree | yes |
|  | H1PF25 | satisfied with your relationship with your father - lots missing. | Categorical frequency: strongly agree, agree, neither agree or disagree, disagree, strongly disagree | yes |
|  | H1PR1 | feel that adults care about you | Categorical ordered: not at all, very little, somewhat, quite a bit, very much, does not apply | yes |
|  | H1PR3 | feel your parents care about you | Categorical ordered: not at all, very little, somewhat, quite a bit, very much, does not apply | yes |
|  | H1PR5 | feel that people in your family home understand you | Categorical ordered: not at all, very little, somewhat, quite a bit, very much, does not apply | yes |
|  | H1PR6 | feel you want to leave home | Categorical ordered: not at all, very little, somewhat, quite a bit, very much, does not apply | yes |
|  | H1PR7 | feel that you and your family have fun together | Categorical ordered: not at all, very little, somewhat, quite a bit, very much, does not apply | yes |
|  | H1PR8 | feel that your family pays attention to you | Categorical ordered: not at all, very little, somewhat, quite a bit, very much, does not apply | yes |
| Stressful life events | H1CO10 | ever been raped - female only- lifetime | Categorical: 0=not raped and female, 1= raped and female, 2=Male and not possible to be raped | yes |
|  | H1NR3 | ever given someone sex in exchange for drugs or money -lifetime | Binary: Yes=1, No=0 | yes |
|  | H1FV1 | saw someone shoot or stab another person - past year | Categorical frequency: never, once, more than once | yes |
|  | H1FV2 | someone pulled a knife or gun on you - past year | Categorical frequency: never, once, more than once | yes |
|  | H1FV3 | someone shot you - past year | Categorical frequency: never, once, more than once | yes |
|  | H1FV4 | someone cut or stabbed you - past year | Categorical frequency: never, once, more than once | yes |
|  | H1FV5 | you got into a physical fight - past year | Categorical frequency: never, once, more than once | yes |
|  | H1FV6 | you were jumped - past year | Categorical frequency: never, once, more than once | yes |
|  | H1FP21_1 | how did pregnancy 1 end -lifetime | Categorical: abortion, live birth, hasn't ended, still birth/miscarriage | yes |
|  | H1FP21_2 | how did pregnancy 2 end -lifetime | Categorical: abortion, live birth, hasn't ended, still birth/miscarriage | yes |
|  | H1FP21_3 | how did pregnancy 3 end -lifetime | Categorical: abortion, live birth, hasn't ended, still birth/miscarriage | yes |
|  | H1SU4 | have any of your friends tried to kill themselves - past 12 months | Binary: Yes=1, No=0 | yes |
|  | H1SU5 | Have any of your friends have succeeded in committing suicide in the past 12 months? | Categorical: yes, no, reported friend attempt but NA outcome, no attempt | yes |
|  | H1SU6 | have any of your family members tried to kill themselves - past 12 months | Binary: Yes=1, No=0 | yes |
|  | H1SU7 | Have any of your family members succeeded in committing suicide in the past 12 months | Categorical: yes, no, reported family attempt but NA outcome, no attempt | yes |
| Family income | PA55 | total family income before taxes - all incomes and sources ($1000) | Numeric: distribution spikes on tens; 0-999 | yes |
|  | PA56 | do you have enough money to pay your bills | Binary: Yes=1, no=0 | yes |
|  | H1GH27I | reason for not seeking healthcare - couldn't pay | Categorical: No to H1GH26, Yes to couldn't pay, No to couldn't pay | yes |
|  | PA59 | how easy/hard is it to get medical care for your family | Categorical ordered: very easy,somewhat easy, somewhat hard, very hard | yes |
| **Built environment** | | | | |
| Healthcare | CAR93870 | Total physicians (non-Federal) providing patient care per 100,000 population - county | Numeric: 0.000 to 1670.517 | no |
|  | CAR90906 | Dentists per 100,000 population - county | Numeric: 0.000 to 190.397 | no |
|  | CAR92918 | Hospitals per 100,000 population - county | Numeric: 0.000 to 23.952 | no |
|  | CAR92948 | Inpatient days in short term general hospitals per capita - county | Numeric: 0.000 to 3.761 | no |
|  | CAG92955 | Presence/absence of hospital abortion provider - county | Binary: 1=yes, 0=no | no |
|  | CAR92953 | Medicaid short term general hospital occupancy rate per 100 beds - county | Numeric: 0.576 to 56.176 | no |
|  | CAG94960 | Family planning service providers per 10,000 population - county | Numeric:0.000 to 3.070 | no |
| Education | C/T/BST90678 | Proportion aged 3 years and over in preprimary school that are enrolled in private school | Numeric:  C: 0.000 to 0.662  T: 0.000 to 1.000  B: 0.000 to 1.000 | no |
|  | C/T/BST90679 | Proportion aged 3 years and over in elementary or high school that are enrolled in private school | Numeric:  C: 0.000 to 0.345  T: 0.000 to 0.844  B: 0.000 to 1.000 | no |
|  | C/T/BST90680 | Proportion aged 25+ with no high school diploma or equivalency | Numeric:  C: 0.052 to 0.611  T: 0.000 to 0.875  B: 0.000 to 0.931 | no |
|  | C/T/BST90686 | Proportion aged 25+ with college degree or more | Numeric:  C: 0.078 to 0.487  T: 0.000 to 0.900  B: 0.000 to 0.944 | no |
|  | C/T/BST90696 | Proportion civilian population aged 16-19 enrolled in school | Numeric:  C: 0.563 to 0.916  T: 0.211 to 1.000  B: 0.000 to 1.000 | no |
|  | C/T/BST90702 | Proportion aged 16-19 not in school or armed forces and not high school graduate | Numeric:  C: 0.010 to 0.309  T: 0.000 to 0.689  B: 0.000 to 0.842 | no |
| Labour force | C/T/BST90714 | Proportion of civilian labor force that is female | Numeric:  C: 0.333 to 0.547  T: 0.182 to 1.000  B: 0.000 to 1.000 | no |
|  | CST90731 | Female labor force opportunity index2 - county | Numeric: 0.026 to 0.091 | no |
|  | C/T/BST90732 | Proportion males (not in the armed forces) aged 16 years and over in the civilian labor force | Numeric:  C: 0.508 to 0.883  T: 0.000 to 0.972  B: 0.000 to 1.000 | no |
|  | C/T/BST90741 | Proportion persons aged 16 years and over in the labor force who are in the armed forces | Numeric:  C: 0.000 to 0.538  T: 0.000 to 0.919  B: 0.000 to 0.919 | no |
|  | C/T/BST90754 | Unemployment rate | Numeric:  C: 0.025 to 0.151  T: 0.000 to 0.658  B:0.000 to 0.593 | no |
|  | C/T/BST90795 | Proportion employed in managerial and professional specialty occupations | Numeric:  C: 0.123 to 0.476  T: 0.000 to 0.749  B: 0.000 to 0.838 | no |
|  | C/T/BST90796 | Proportion employed in technical, sales and administrative support occupations | Numeric:  C: 0.163 to 0.384  T: 0.099 to 0.511  B: 0.000 to 0.663 | no |
|  | C/T/BST90797 | Proportion employed in service occupations | Numeric:  C: 0.079 to 0.264  T: 0.000 to 0.560  B: 0.000 to 0.607 | no |
|  | C/T/BST90798 | Proportion employed in farming, forestry, and fishing occupations | Numeric:  C: 0.002 to 0.256  T: 0.000 to 0.371  B: 0.000 to 0.530 | no |
|  | C/T/BST90799 | Proportion employed in precision production, craft, and repair occupations | Numeric:  C: 0.034 to 0.283  T: 0.000 to 0.347  B: 0.000 to 0.517 | no |
|  | C/T/BST90800 | Proportion employed as operators, fabricators, and laborers | Numeric:  C: 0.058 to 0.449  T: 0.000 to 0.536  B: 0.000 to 0.918 | no |
|  | W1PCC190 | Proportion working outside the county of  residence, 1990 | NOTE: Smallest 5 and largest 5 values are displayed  Numeric:  Small: 0.017 to 0.030  Large: 0.698 to 0.738 | no |
|  | W1PCC290 | Median travel time to work, 1990 | Neumeric:  2 = 5–9 minutes, 3 = 10–14 minutes, 4 = 15–19 minutes, 5 = 20–24 minutes, 6 = 25–29 minutes, 7 = 30–34 minutes, 8 = 35–39 minutes, 9 = 40–44 minutes, and 10 = 45–59 minutes | no |
| Crime | CUC93982 | Total crime rate per 100,000 population in reporting area - county | Numeric: 0.000 to 16855.295 | no |
|  | CUC93990 | Total juvenile arrests per 100,000 population in reporting area - county | Numeric: 0.000 to 2310.586 | no |
|  | CUC93998 | Total adult arrests per 100,000 population in reporting area - county | Numeric: 0.000 to 9403.547 | no |
| Religion | CCM90A07 | Church adherents per capita - county | Numeric: 0.088 to 1.082 | no |
|  | CCM90A14 | Dispersion in religious composition (Jewish, Catholic, Black Baptist, “other”conservative, “other”moderate, “other”liberal) - county | Numeric: 0.059 to 0.961 | no |
| Socioeconomic and Poverty | CAR90404 | Crude death rate (per 1,000 individuals) - county | Numeric: 2.649 to 18.837 | no |
|  | C/T/BST90431 | Proportion persons that are in emergency shelters for homeless persons or visible in street locations | Numeric:  C: 0.000 to 0.012  T: 0.000 to 0.472  B: 0.000 to 0.570 | no |
|  | C/T/BST90544 | Median household income | Numeric:  C: $12732.000 to $54628.000  T: $4999.000 to $125053.000  B: $4999.000 to $148752.000 | no |
|  | C/T/BST90550 | Standard deviation in household income distribution | Numeric:  C: $15944.650 to $49712.209  T: $6916.090 to $66828.276  B: $5509.304 to $71297.573 | no |
|  | C/T/BST90584 | Proportion families with income less than $15,000 | Numeric:  C: 0.039 to 0.508  T: 0.000 to 0.903  B: 0.000 to 0.947 | no |
|  | C/T/BST90591 | Per capita income in 1989 | Numeric:  C: $6084.000 to $27862.000  T: $1838.000 to $68822.000  B: $1603.000 to $136442.000 | no |
|  | C/T/BST90598 | Proportion persons with income in 1989 below poverty level | Numeric:  C: 0.022 to 0.402  T: 0.000 to 0.864  B: 0.000 to 0.861 | no |
|  | C/T/BST90626 | Proportion families with income in 1989 below poverty level | Numeric:  C: 0.014 to 0.359  T: 0.000 to 0.859  B: 0.000 to 0.859 | no |
|  | C/T/BST90674 | Proportion related children under 18 years in families with income in 1989 below poverty level | Numeric:  C: 0.019 to 0.526  T: 0.000 to 1.000  B: 0.000 to 1.000 | no |
|  | C/T/BST90816 | Median gross rent of specified renter-occupied housing units paying cash rent | Numeric:  C: $192.000 to $790.000  T: $99.000 to $1001.000  B: $99.000 to $1001.000 | no |
|  | C/T/BST90822 | Standard deviation in gross rent of specified renter-occupied housing units paying cash rent | Numeric:  C: $86.001 to $355.855  T: $58.979 to $440.570  B: $0.000 to $440.570 | no |
|  | C/T/BST90803 | Proportion occupied housing units that are owner-occupied | Numeric:  C: 0.179 to 0.860  T: 0.000 to 0.985  B: 0.000 to 1.000 | no |
|  | C/T/BST90857 | Proportion occupied housing units lacking complete plumbing facilities | Numeric:  C: 0.002 to 0.114  T: 0.000 to 0.297  B: 0.000 to 0.292 | no |
|  | C/T/BST90865 | Proportion housing units lacking complete kitchen facilities | Numeric:  C: 0.002 to 0.084  T: 0.000 to 0.221  B: 0.000 to 0.449 | no |
|  | CSD90868 | Proportion school age children (6-19 years) who are “at risk” school age children - county | Numeric: 0.002 to 0.247 | no |
|  | CSD90869 | Proportion pre-school age children (4-5 years) who are “at risk” pre-school age children - county | Numeric: 0.000 to 0.246 | no |
| Political climate | CCU92A27 | Proportion voting democratic in 1992 presidential election - county | Numeric: 0.222 to 0.783 | no |
|  | CCU92A28 | Proportion voting republican in 1992 presidential election - county | Numeric: 0.159 to 0.643 | no |
|  | CCU92A29 | Proportion voting for Perot in 1992 presidential election - county | Numeric: 0.050 to 0.328 | no |
|  | CCU87A54 | Proportion of local government direct general expenditures for education - county | Numeric: 0.003 to 0.803 | no |
|  | CCU87A55 | Proportion of local government direct general expenditures for health and hospitals - county | Numeric: 0.000 to 0.480 | no |
|  | CCU87A56 | Proportion of local government direct general expenditures for public welfare - county | Numeric: 0.000 to 0.181 | no |
|  | CCU87A57 | Proportion of local government direct general expenditures for police protection - county | Numeric: 0.006 to 0.114 | no |
|  | CCU87A58 | Proportion of local government direct general expenditures, other  type - county | Numeric: 0.109 to 0.875 | no |
|  | W1PCP192 | Proportion of votes cast for the Democratic  presidential candidate, 1992. | Numeric: 0.222 to 0.783 | no |
|  | W1PCP292 | Proportion of votes cast for the Republican  presidential candidate, 1992. | Numeric: 0.159 to 0.643 | no |
| **Natural environment** | | | | |
| Population density | C/T/BST90001 | Total population (county) | Numeric:  C: 4279 to 8863164  T: 8 to 71872  B: 0 to 34873 | no |
|  | C/T/BST90005 | Density (persons/sq. km.) (county) | Numeric:  C: 0.000 to 20.239  T: 0.000 to 69.172  B: 0.000 to 87.516 | no |
|  | C/T/BST90437 | Households (absolute number) | Numeric:  C: 1793.000 to 2994343.000  T: 0.000 to 29261.000  B: 0.000 to 10672.000 | no |
|  | W1CN01_8 | Road connectivity (Alpha index, 8km) | Numeric: 0.0497 to 0.5371 | no |
|  | W1CN04_8 | Cul de sac density-sqkm, 8km | Numeric: 0.00491600536558 to 15.2740286708819 | no |
|  | W1CN13_8 | Link (roads) count, 8km | Numeric: 26 to 25862 | no |
|  | W1P90_P8 | 1990 block group population, 8 km | Numeric: 7.2 to 2783844.2 | no |
| Urban distances | URBAN | Urban Rural Code for Block Group Residence | Binary: 1=Completely Urbanized, 0=Partly Rural | no |
|  | W1PKC1K8 | Park count 8km | Numeric: 0 to 195 | no |
|  | W1RU1 | Rural-Urban commuting area (RUCA) Code, 1990 | Categorical: 1 to 10.5 | no |
|  | W1SD1 | Distance to school in meters | Numeric: 0 to 120054 | no |
| Climate | W1CL1_A | Mean total precipitation, annually. Reported in inches | Numeric: 3.11 to 91 | no |
|  | W1CL2_A | Mean total snowfall, annually. Reported in inches | Numeric: 0 to 150.9 | no |
|  | W1CL3_A | Mean daytime sky cover, annually. Reported as percent sky cover | Numeric: 30 to 80 | no |
|  | W1CL4_A | Mean sunshine total hours, annually. Reported as mean monthly hours | Numeric: 1488 to 4015 | no |
|  | W1CL7_A | Mean number of days maximum temperature >= 90 F, annually | Numeric: 0 to 175 | no |
|  | W1CL8_A | Mean number of days minimum temperature <= 32 F, annually | Numeric: 0 to 257 | no |
|  | W1LCA_L8 | Landscape total area (sq m) | Numeric: 106832700 to 203444100 | no |

| **Table 3. Data selection process across the four data sources** | | |
| --- | --- | --- |
|  | Raw N vars | Selected N vars |
| INHOME | 2525 | 86 |
| Context | 2682 | 57 |
| Obesity and Neighborhood Environment Files | 274 | 14 |
| Political | 16 | 4 |

## *1C. Missingness for predictors*

We assessed the proportion of true missing data across all variables. Missingness may be informative for some variables, therefore, for categorical variables that participants responded to in the questionnaire, responses of “Don’t know”, “Not applicable”, “Legitimate skip”, and “Refused” were all included in the matrices as dummy coded responses. Where we had data coming from different sources - parent questionnaire data or geocoded data - and data were empty (true missing) for some people, we used random-sample imputation. Random-sample imputation works by replacing missing values with randomly sampled observed values from the same variable. Thus, missing values were replaced by random draws from the observed distribution of each variable (sampling with replacement), preserving the marginal distribution but not modelling relationships between variables. This imputation method is simple and relies on the randomness of the missingness. However, this approach also preserves the observed distribution, works across continuous and categorical variables, is computationally efficient, avoids any deterministic imputation (e.g. like mean imputation), and does not assume a parametric distribution.

| **Table 4**. Predictors with true missing values | | | | | | | |
| --- | --- | --- | --- | --- | --- | --- | --- |
| **Variable** | **N missing** | **Variable** | **N missing** | **Variable** | **N missing** | **Variable** | **N missing** |
| PA55 | 545 | BST90797 | 66 | BST90626 | 86 | H1FS1 | 1 |
| PA56 | 571 | BST90798 | 66 | BST90674 | 127 | H1FS3 | 1 |
| PA59 | 492 | BST90799 | 66 | CST90816 | 40 | H1FS8 | 4 |
| CAR93870 | 40 | BST90800 | 66 | CST90822 | 40 | H1FS10 | 1 |
| CAR90906 | 40 | CUC93982 | 275 | BST90803 | 70 | H1FS12 | 1 |
| CAR92918 | 40 | CUC93990 | 380 | BST90857 | 70 | H1FS13 | 1 |
| CAR92948 | 40 | CUC93998 | 380 | BST90865 | 70 | H1FS19 | 2 |
| CAG92955 | 40 | CCM90A07 | 40 | CSD90868 | 40 | H4MH18 | 1 |
| CAR92953 | 331 | CCM90A14 | 40 | CSD90869 | 40 | H4MH19 | 1 |
| CAG94960 | 40 | CAR90404 | 40 | CCU92A27 | 40 | H4MH20 | 1 |
| CST90678 | 49 | BST90431 | 63 | CCU92A28 | 40 | H4MH24 | 1 |
| BST90679 | 246 | BST90544 | 198 | CCU92A29 | 40 | H4MH25 | 1 |
| BST90680 | 65 | BST90550 | 198 | CCU87A54 | 40 | H4MH27 | 1 |
| BST90686 | 65 | BST90584 | 86 | CCU87A55 | 40 | URBAN | 60 |
| CST90696 | 40 | BST90591 | 68 | CCU87A56 | 40 | W1LCA_L8 | 14 |
| CST90702 | 40 | BST90598 | 63 | CCU87A57 | 40 | W1CL2_A | 14 |
| BST90714 | 66 | BST90754 | 125 | CCU87A58 | 40 | BST90005 | 60 |
| CST90731 | 40 | BST90795 | 66 | BST90001 | 60 | BST90437 | 60 |
| BST90732 | 72 | BST90796 | 66 | BST90741 | 66 |  |  |

## *1D. Statistical analyses*

We computed linear mixed models (LMM) to estimate the contribution of environmental and genetic similarity to depression symptoms in three steps. First, we prepared the environmental data by selecting the numeric variables, addressing missingness, and dummy coding each variable with the reference categories reflecting "healthy" or "typical". We then applied a principal component transformation to reduce multicollinearity among the environmental variables. To do this, eigenvalue decomposition of a covariance matrix across the selected variables was performed to extract the eigenvectors. The original environmental matrix was then multiplied by the eigenvector matrix to generate an orthogonal set of transformed variables. These scores were standardized and scaled by 1/sqrt(𝑚), where 𝑚 is the number of variables, to produce a normalized set of transformed exposures for subsequent analysis. Second, we used the -pdmx function within the mgt2 computer programme to create an N x N relatedness matrix structured by the similarity on the transformed environmental variables. Mtg2 is a software for fitting linear mixed models with multiple relationship matrices. Third, we use mgt2 to include this matrix (and any others included in the model) as a random effect in a linear model. Environmental and genetic variance components (–g) were estimated using a single-trait REML model. Phenotype files for each wave were analysed separately while adjusting for categorical and continuous covariates provided through the –cc and –qc arguments, respectively. To express variance components as proportions of the total phenotypic variance, mtg2’s delta method (–delta2) was applied to calculate the derived quantities and their standard errors using the delta method. All mtg2 analyses were executed on a high-performance computing cluster at Purdue University (Rossmann) using batch scripts to iterate across outcome waves. All scripts are provided on GitHub: <https://github.com/knthompson26/findmedep>.

**Table 5** shows the model specification for each set of analyses. 𝐘_N,1_ is the depression symptom outcome with N individuals, 𝑿_N,c_ is a matrix of covariates (year of birth, sex, and 20 ancestry principal components), 𝛃_c,1_ is a vector of fixed effects, 𝐬 and $g$ are vectors of random effects (**s** for “social” and **g** for “genetic”) and 𝜺 represents error. We assume the random effects follow multivariate normal distributions: $s\sim N(0, S\sigma_{s}^{2})$ and $g\sim N(0,A\sigma_{g}^{2})$, where $A$ and $S$ are the environmental and genetic relatedness matrices. Off-diagonals of the relatedness matrices reflect the similarity (variance-covariance) between two individuals. The residuals follow $\varepsilon\sim N(0, I\sigma_{e}^{2})$ where $I$ is an identity matrix and the error terms are independent and identically distributed.

The genetic relatedness matrix $A$ uses single nucleotide polymorphism data to capture genome-wide similarity and was generated using GCTA. We computed $S$ using the computer programme mtg2 to calculate the similarity between each pair of individuals, $i$ and $j$, as s_i,j_ = $\frac{\sum_{1}^{M} z_{i,m}z_{j,m}}{M}$, where $z_{i,m}$ and $z_{j,m}$are the centred and standardised values of the variable $m$ for individual $i$ and $j$, and $M$ is the total number of measures. In matrix form: $S = \frac{ZZ'}{M}$ , where $Z$_N,M_ contains the standardised variables for all individuals.

The total variance in depression is decomposed as: $\sigma_{dep}^{2}= \sigma_{s}^{2} +\sigma_{g}^{2}+\sigma_{e}^{2}$. The proportion of variance explained by environmental similarity is: $R^{2}= \frac{\sigma_{s}^{2}}{\sigma_{g}^{2} + \sigma_{s}^{2} + \sigma_{e}^{2}}$ which quantifies the contribution of the shared environment over and above genetic similarity and individual error.

To estimate covariance between random effects, we extended the variance-components model to allow the similarity structures underlying each domain to covary. Covariance between two domains or systems was estimated by constructing cross-product kernels between the Cholesky factors of their relatedness matrices, which capture the degree to which individuals who are similar in one domain also tend to be similar in another. These covariance kernels were included alongside the main-effect kernels in the mixed model, allowing the variance explained by each domain and the covariance between domains to be estimated simultaneously using restricted maximum likelihood in MTG2. This decomposes phenotypic variance into domain/system-specific variance components and covariance components reflecting shared structure between domains.

The interaction matrix (𝗴*𝐬) was computed as the elementwise Hadamard product of the untransformed environmental matrix and genomic relatedness matrix. Each pairwise entry reflects the extent to which two individuals are simultaneously similar genetically and similar environmentally. This interaction kernel was included as an additional random effect alongside the main GRM and ERM in a linear mixed model estimated by REML in mtg2. The interaction variance component quantifies phenotypic variation attributable to genetic effects that differ across environmental similarity patterns, beyond the additive contributions of genetic and environmental effects.

| **Table 5.** Model equation and specification to assess the influence of large-scale risk factors to depression symptoms | |
| --- | --- |
| **Equation** | |
| How do prominent risk factors for depression contribute to symptom occurrence? | |
|  | Broad environmental and genetic influences |
| 1 | $Y = X\beta+ s +\varepsilon$ |
| 2 | $Y = X\beta+ g +\varepsilon$ |
| 3 | $Y = X\beta+ s +g+\varepsilon$ |
| 4 | $Y = X\beta+ s +g+\varepsilon, \sigma_{sg}⧣ 0$ |
| 5 | $Y = X\beta+ s +g + g*s+\varepsilon$ |
| 6 | $Y = X\beta+ s +g + g*s+\varepsilon, \sigma_{sg}⧣ 0$ |
|  | Domain-specific influences |
| 7 | $Y = X\beta+ g +s1 +s2 +s3 ... +\varepsilon$ |
| 8 | $Y = X\beta+g + s1 +s2 +s3 ... +\varepsilon, \sigma_{ss}⧣ 0$ |
| 9 | $Y = X\beta+g + s1 +s2 +s3 ...+ s1 *s2+ ...+\varepsilon$ |
| 10 | $Y = X\beta+g + s1 +s2 +s3 ...+ s1 *s2+ ... +\varepsilon, \sigma_{ss}⧣ 0$ |
| To what extent can large-scale environmental data explain depression symptoms? | |
| 11 | $Y = X\beta+bio +psych +social+built +natural +\varepsilon$ |
| 12 | $Y = X\beta+bio+psych +social+built +natural +\varepsilon, \sigma_{ss}⧣ 0$ |
| 13 | $Y = X\beta+bio+psych+social+built+natural+social*built +\varepsilon$ |

# Supplement 2. Results

## *Descriptive statistics*

**Table 6** provides the correlations between each depression symptom score (W1A, W1B, W2, and W4). **Table 7** displays the descriptive statistics for each relatedness matrix before being entered as a random effect in the linear mixed model. **Table 8** shows the correlations between each domain matrix and **Table 9** shows the correlations between each system matrix.

*All results are provided with four depression scores: W1A, the full CES-D scale at Wave 1; W1B, the reduced CES-D score at Wave 1 to match items asked at subsequent waves; W2, reduced CES-D score at Wave 2; W4, reduced CES-D score at Wave 4.* ***W1B is reported in the main manuscript text to aid comparison across all Waves.***

| **Table 6**. Correlations between each depression symptom score | | | | |
| --- | --- | --- | --- | --- |
|  | **W1A** | **W1B** | **W2** | **W4** |
| **W1A** | 1.000 | 0.963 | 0.580 | 0.323 |
| **W1B** | 0.963 | 1.000 | 0.572 | 0.312 |
| **W2** | 0.580 | 0.572 | 1.000 | 0.337 |
| **W4** | 0.323 | 0.312 | 0.337 | 1.000 |

| **Table 7.** Descriptive statistics for each relatedness matrix | | | | | | | | |
| --- | --- | --- | --- | --- | --- | --- | --- | --- |
| **Relatedness matrix** | **N** | **Mean** | **Median** | **SD** | **Min** | **Max** | **Proportion similar** | **Diagonal mean** |
| *Domains* | | | | | | | | |
| Demographic | 3867 | 0.000 | -0.027 | 0.215 | -12.854 | 96.392 | 0.368 | 0.985 |
| Friends and family | 3867 | 0.000 | -0.006 | 0.092 | -83.716 | 44.688 | 0.445 | 0.944 |
| Positive cognition | 3867 | 0.000 | -0.033 | 0.204 | -218.454 | 115.838 | 0.386 | 1.106 |
| School | 3867 | 0.000 | -0.014 | 0.109 | -45.691 | 9.849 | 0.429 | 0.816 |
| Stressful life events | 3867 | 0.019 | 0.014 | 0.106 | -42.267 | 44.269 | 0.658 | 0.893 |
| General health | 3867 | 0.000 | -0.020 | 0.205 | -37.743 | 95.411 | 0.376 | 0.936 |
| Mental health | 3867 | 0.000 | -0.021 | 0.176 | -2.076 | 118.249 | 0.437 | 0.861 |
| Physical health | 3867 | 0.000 | -0.010 | 0.110 | -18.053 | 41.820 | 0.435 | 0.999 |
| *Systems* | | | | | | | | |
| Psychological | 3867 | 0 | -0.005 | 0.072 | -12.697 | 17.215 | 0.455 | 1 |
| Social | 3867 | 0 | -0.004 | 0.136 | -4.909 | 190.492 | 0.457 | 1 |
| Built | 3867 | 0 | -0.017 | 0.169 | -21.734 | 20.072 | 0.427 | 1 |
| Natural | 3867 | 0 | -0.026 | 0.257 | -2.489 | 19.166 | 0.441 | 1 |
| **Note**. As an example for how to interpret the “Proportion similar”: For the psychological system, 45.5% of all pairs of individuals are more similar than the sample average. When the “Diagonal mean” = 1, this means the matrix was fully standardised. | | | | | | | | |

| **Table 8.** Correlations across each of the domain relatedness matrices | | | | | | | | |
| --- | --- | --- | --- | --- | --- | --- | --- | --- |
|  | **Demographic** | **Family Relationships** | **Positive Cognition** | **School** | **Stressful Life Events** | **General Health** | **Mental Health** | **Physical Health** |
| **Demographic** | 1.000 | 0.021 | 0.008 | 0.010 | 0.013 | 0.058 | 0.006 | 0.004 |
| **Family Relationships** | 0.021 | 1.000 | 0.014 | 0.009 | 0.006 | 0.003 | 0.004 | 0.003 |
| **Positive Cognition** | 0.008 | 0.014 | 1.000 | 0.012 | 0.002 | 0.007 | 0.007 | 0.007 |
| **School** | 0.010 | 0.009 | 0.012 | 1.000 | 0.006 | 0.007 | 0.010 | 0.008 |
| **Stressful Life Events** | 0.013 | 0.006 | 0.002 | 0.006 | 1.000 | 0.003 | 0.005 | 0.004 |
| **General Health** | 0.058 | 0.003 | 0.007 | 0.007 | 0.003 | 1.000 | 0.012 | 0.009 |
| **Mental Health** | 0.006 | 0.004 | 0.007 | 0.010 | 0.005 | 0.012 | 1.000 | 0.023 |
| **Physical Health** | 0.004 | 0.003 | 0.007 | 0.008 | 0.004 | 0.009 | 0.023 | 1.000 |

| **Table 9**. Correlations across each of the system relatedness matrices | | | | |
| --- | --- | --- | --- | --- |
|  | **Psychological** | **Social** | **Built** | **Natural** |
| **Psychological** | 1.000 | 0.025 | 0.002 | 0.002 |
| **Social** | 0.025 | 1.000 | 0.001 | 0.002 |
| **Built** | 0.002 | 0.001 | 1.000 | 0.268 |
| **Natural** | 0.002 | 0.002 | 0.268 | 1.000 |

## *2A. Broad environmental and genetic influences on depression symptoms*

We first split the 81 prominent individual-level factors into genetic data (represented as 1 variable) and non-genetic data, which we call “environmental factors” even though some have a genetic component (80 variables). Estimates from linear mixed models were comparable to linear regression estimates using all environmental variables (W1A adjR^2^=0.55; W1B adjR^2^=0.51; W2 adjR^2^=0.31; W4 adjR^2^=0.13). However, variance components represent the proportion of phenotypic variance attributable to structured similarity captured by each random-effect matrix, and should not be interpreted as model R² from regression. Unlike R² from fixed-effect regression, variance components reflect population-level covariance partitioning rather than predictive variance explained.

*All results are provided with four depression scores: W1A, the full CES-D scale at Wave 1; W1B, the reduced CES-D score at Wave 1 to match items asked at subsequent waves; W2, reduced CES-D score at Wave 2; W4, reduced CES-D score at Wave 4.* ***W1B is reported in the main manuscript text to aid comparison across all Waves.*** *Out-of-bounds components are ones in which estimation resulted in negative variances, missing standard errors, or computed correlations that were +/-1.*

| **Table 10.** Parameter values for broad environmental and genetic influences on depression symptoms | | | | |
| --- | --- | --- | --- | --- |
| **Input** | **W1A** | **W1B** | **W2** | **W4** |
| **Total environment** $Y = X\beta+ s+\varepsilon$ | | | | |
| Environment | 0.592 (0.021)* | 0.551 (0.022)* | 0.341 (0.022)* | 0.136 (0.016)* |
| Error | 0.408 (0.021)* | 0.449 (0.022)* | 0.659 (0.022)* | 0.864 (0.016)* |
| Total variance explained | 0.592 | 0.551 | 0.341 | 0.136 |
| **Genetic** $Y = X\beta+ g+\varepsilon$ | | | | |
| Genetic | 0.266 (0.095)* | 0.261 (0.094)* | 0.162 (0.095) | 0.25 (0.095)* |
| Error | 0.734 (0.095)* | 0.739 (0.094)* | 0.838 (0.095)* | 0.75 (0.095)* |
| Total variance explained | 0.266 | 0.261 | 0.162 | 0.25 |
| **Total environment and genetic** $Y = X\beta+ s + g+\varepsilon$ | | | | |
| Genetic | 0.039 (0.038) | 0.036 (0.043) | 0.017 (0.062) | 0.152 (0.083) |
| Environment | 0.591 (0.021)* | 0.55 (0.022)* | 0.34 (0.022)* | 0.135 (0.016)* |
| Error | 0.37 (0.043)* | 0.414 (0.047)* | 0.642 (0.066)* | 0.713 (0.083)* |
| Total variance explained | 0.63 | 0.586 | 0.358 | 0.287 |
| **Total environment and genetic, rGE** $Y = X\beta+ s + g+\varepsilon$ **,** $\sigma_{sg}$⧣ 0 | | | | |
| Genetic | 0.047 (0.043) | 0.043 (0.047) | 0.018 (0.067) | 0.156 (0.085) |
| Environment | 0.663 (0.051)* | 0.614 (0.049)* | 0.382 (0.037)* | 0.139 (0.02)* |
| Error | 0.412 (0.053)* | 0.459 (0.058)* | 0.709 (0.08)* | 0.725 (0.09)* |
| Covariance | -0.1219 | -0.1162 | -0.1089 | -0.0203 |
| Genetic ~ Environment | r=-0.346 (0.238) | r=-0.358 (0.278) | r=-0.663 (1.309) | r=-0.069 (0.18) |
| Total variance explained | 0.588 | 0.541 | 0.291 | 0.275 |
| **Total environment and genetic, GxE** $Y = X\beta+ s + g+s*g+\varepsilon$ | | | | |
| Genetic | 0.025 (0.03) | 0.018 (0.034) | 0.014 (0.052) | 0.146 (0.076) |
| Environment | 0.524 (0.024)* | 0.494 (0.024)* | 0.306 (0.023)* | 0.113 (0.016)* |
| Genetic x Environment | 0.351 (0.029)* | 0.343 (0.03)* | 0.363 (0.034)* | 0.265 (0.038)* |
| Error | 0.1 (0.034)* | 0.145 (0.038)* | 0.316 (0.059)* | 0.475 (0.083)* |
| Total variance explained | 0.9 | 0.855 | 0.684 | 0.525 |
| **Total environment and genetic, rGE and GxE** $Y = X\beta+ s + g+s*g+\varepsilon$ **,** $\sigma_{sg}$⧣ 0 | | | | |
| Genetic | 0.028 (0.032) | 0.02 (0.035) | 0.016 (0.055) | 0.149 (0.077) |
| Environment | 0.55 (0.041)* | 0.525 (0.041)* | 0.336 (0.034)* | 0.116 (0.019)* |
| Genetic x Environment | 0.363 (0.033)* | 0.359 (0.035)* | 0.389 (0.04)* | 0.268 (0.04)* |
| Error | 0.108 (0.036)* | 0.157 (0.042)* | 0.345 (0.065)* | 0.48 (0.086)* |
| Covariance | -0.0489 | -0.0605 | -0.0867 | -0.0119 |
| Genetic ~ Environment | r=-0.198 (0.247) | r=-0.294 (0.368) | r=-0.584 (1.034) | r=-0.045 (0.194) |
| Total variance explained | 0.892 | 0.843 | 0.655 | 0.52 |
| **Note.** Standardised variance component (standard error) for each depression symptom outcome (W1A, W1B, W2, W4). Correlation coefficients provided for models including covariance parameters. *indicates p value < 0.05. Out-of-bounds components are ones in which estimation resulted in negative variances, missing standard errors, or computed correlations that were +/-1. | | | | |

### 2A.1 Sensitivity analysis: Top 10 heritable factors removed

To assess the genetic contribution to each environmental item included in the environmental relatedness matrix, SNP-based heritability was estimated for all dummy-coded variables individually. Variables with no variation across individuals were excluded. SNP-based heritability was estimated using GCTA REML. Models adjusted for categorical and continuous covariates, and prevalence estimates were supplied for binary traits to obtain liability-scale estimates. Heritability estimates and standard errors were extracted from output files, and 95% confidence intervals were computed to identify statistically significant estimates (**Figure 1**). The highest factor heritability before exclusion was 43.87% and after exclusion was 34.80%. We found no substantial difference in genetic, environment, and interaction components across the two sets of models (**Table 11**).

**Figure 1.** Heritability estimates for all environmental items.

10 most heritable factors:

1. H1GH28_numeric_raw_dummy_8: Perceived weight
2. H1GH48_numeric_raw_dummy_6: health/emotion problem day off
3. H1GH49_numeric_raw_dummy_8: Missed recreation due to health/emotion
4. H1PF35_numeric_raw_dummy_3: you feel socially accepted
5. H1HS3_numeric_raw_dummy_1: not had counselling
6. H1PR7_numeric_raw_dummy_4: family have fun
7. H1PR7_numeric_raw_dummy_2: family have fun
8. H1GH48_numeric_raw_dummy_4: health/emotion problem day off
9. H1GH8_numeric_raw_dummy_8: Fatigue
10. H1GH17_numeric_raw_dummy_1: Poor appetite

| **Table 11.** Parameter values for broad environmental and genetic influences on depression symptoms - top 10 heritable items removed | | | | |
| --- | --- | --- | --- | --- |
| **Input** | **W1A** | **W1B** | **W2** | **W4** |
| **Total environment and genetic** $Y = X\beta+ s + g+\varepsilon$ | | | | |
| Genetic | 0.0431 (0.0388) | 0.0413 (0.0428) | 0.0142 (0.0619) | 0.1526 (0.0826) |
| Environment | 0.5869 (0.0216)* | 0.5463 (0.0223)* | 0.3394 (0.0224)* | 0.1358 (0.0161)* |
| Error | 0.3701 (0.0431)* | 0.4124 (0.047)* | 0.6464 (0.0655)* | 0.7116 (0.0833)* |
| Total variance explained | 0.6299 | 0.5876 | 0.3536 | 0.2884 |
| **Total environment and genetic, GxE** $Y = X\beta+ s + g+s*g+\varepsilon$ | | | | |
| Genetic | 0.0295 (0.0305) | 0.0232 (0.0339) | 0.0168 (0.0516) | 0.1461 (0.0759) |
| Environment | 0.5222 (0.0244)* | 0.492 (0.0247)* | 0.3073 (0.0234)* | 0.1147 (0.0163)* |
| Genetic x Environment | 0.3493 (0.0288)* | 0.3403 (0.0298)* | 0.3566 (0.0341)* | 0.2638 (0.038)* |
| Error | 0.0991 (0.0343)* | 0.1445 (0.0385)* | 0.3193 (0.0586)* | 0.4755 (0.0824)* |
| Total variance explained | 0.9009 | 0.8555 | 0.6807 | 0.5245 |
| **Note.** Standardised variance component (standard error) for each depression symptom outcome (W1A, W1B, W2, W4); *indicates p value < 0.05. Out-of-bounds components are ones in which estimation resulted in negative variances, missing standard errors, or computed correlations that were +/-1. | | | | |

### 2A.2 Sensitivity analysis: Health factors removed

Health items that are removed across general, mental, and physical health domains: H1GH1_raw, H1PL1_raw, H1GH2_raw, H1GH3_raw, H1GH4_raw, H1GH5_raw, H1GH6_raw, H1GH7_raw, H1GH8_raw, H1GH9_raw, H1GH10_raw, H1GH11_raw, H1GH12_raw, H1GH13_raw, H1GH14_raw, H1GH15_raw, H1GH16_raw, H1GH17_raw, H1GH18_raw, H1GH19_raw, H1GH22_raw, H1GH26_raw, H1GH28_raw, H1GH29_raw, H1HS3_raw, H1GH48_raw, H1GH49_raw.

We found no substantial difference in genetic, environment, and interaction components across the two sets of models (**Table 12**).

| **Table 12.** Parameter values for broad environmental and genetic influences on depression symptoms - health domains removed | | | | |
| --- | --- | --- | --- | --- |
| **Input** | **W1A** | **W1B** | **W2** | **W4** |
| **Total environment and genetic** $Y = X\beta+ s + g+\varepsilon$ | | | | |
| Genetic | 0.0852 (0.0467) | 0.0815 (0.0495) | 0.0607 (0.0658) | 0.1627 (0.0846) |
| Environment | 0.5058 (0.0263)* | 0.4748 (0.0265)* | 0.291 (0.0239)* | 0.1053 (0.015)* |
| Error | 0.4091 (0.051)* | 0.4437 (0.0538)* | 0.6483 (0.0692)* | 0.732 (0.0852)* |
| Total variance explained | 0.5909 | 0.5563 | 0.3517 | 0.268 |
| **Total environment and genetic, GxE** $Y = X\beta+ s + g+s*g+\varepsilon$ | | | | |
| Genetic | 0.0563 (0.0353) | 0.0571 (0.0382) | 0.0607 (0.0553) | 0.1488 (0.0788) |
| Environment | 0.4463 (0.0286)* | 0.426 (0.0285)* | 0.2728 (0.0252)* | 0.0933 (0.0154)* |
| Genetic x Environment | 0.3697 (0.0314)* | 0.3529 (0.0318)* | 0.3134 (0.0339)* | 0.2013 (0.0359)* |
| Error | 0.1276 (0.0391)* | 0.164 (0.0426)* | 0.3531 (0.0621)* | 0.5567 (0.0844)* |
| Total variance explained | 0.8724 | 0.836 | 0.6469 | 0.4433 |
| **Note.** Standardised variance component (standard error) for each depression symptom outcome (W1A, W1B, W2, W4); *indicates p value < 0.05. Out-of-bounds components are ones in which estimation resulted in negative variances, missing standard errors, or computed correlations that were +/-1. | | | | |

## *2B. Domain specific influences on depression symptoms*

To disentangle which factors contribute the most to depression symptoms, we partitioned the 81 factors into interview-specific domains of demographics, relationships with friends and family, physical health, mental health, general health, feelings about personal abilities and surroundings which we termed positive cognition, school factors, stressful life events, and genetic influence. We included each domain as a separate random effect in the linear mixed model to assess how much variance in depression symptoms is uniquely explained by each domain. We were not able to estimate all domain-by-domain covariances accurately due to the number of random effects that would need to be included in the model. For the domain-by-domain interaction models, we only modeled gene-by-environment interaction components (**shown in Figure 2 below**).

| **Table 13.** Parameter values for domain influences on depression symptoms | | | | |
| --- | --- | --- | --- | --- |
| **Input** | **W1A** | **W1B** | **W2** | **W4** |
| **Domains** $Y = X\beta+ sdem +sphy +smen+sgen+ssch+sfrf+spos+ssle +\varepsilon$ | | | | |
| Demographics | 0.0038 (0.0025) | 0.003 (0.0023) | 0.0136 (0.0062)* | 0.0135 (0.0061)* |
| Friends and family | 0.0278 (0.0075)* | 0.029 (0.0078)* | 0.0216 (0.0073)* | 0.0081 (0.0054) |
| Positive cognition | 0.1705 (0.0447)* | 0.1646 (0.0436)* | 0.0718 (0.0223)* | 0.016 (0.007)* |
| School | 0.0526 (0.0138)* | 0.0433 (0.0119)* | 0.0177 (0.0068)* | 0.0083 (0.0051) |
| Stressful life events | 0.0038 (0.0029) | 5e-04 (0.002) | 0.0086 (0.0046) | 0.0119 (0.0056)* |
| General health | 0.0111 (0.0052)* | 0.011 (0.0053)* | 0.0085 (0.0046) | 0.0063 (0.004) |
| Mental health | 0.1028 (0.0318)* | 0.0999 (0.031)* | 0.0797 (0.0255)* | 0.0245 (0.01)* |
| Physical health | 0.0442 (0.0101)* | 0.0334 (0.0084)* | 0.0096 (0.0047)* | 0.0158 (0.0061)* |
| Genetic | 0.0674 (0.0543) | 0.0594 (0.0574) | 0.014 (0.0702) | 0.1305 (0.0839) |
| Error | 0.516 (0.0637)* | 0.5559 (0.0671)* | 0.7547 (0.0757)* | 0.765 (0.0847)* |
| Total variance explained | 0.484 | 0.4441 | 0.2453 | 0.235 |
| **Domains, rD** $Y = X\beta+ g +sdem +sphy +smen+sgen+ssch+sfrf+spos+ssle +\varepsilon$**,** $\sigma_{s,s}$⧣ 0 | | | | |
| Demographics | 0.0044 (0.0021)* | 0.0038 (0.002) | 0.0096 (0.0032)* | 0.0083 (0.0031)* |
| Friends and family | 0.027 (0.0066)* | 0.0248 (0.0063)* | 0.0185 (0.0055)* | 0.0083 (0.0044) |
| Positive cognition | 0.0537 (0.0106)* | 0.0496 (0.0097)* | 0.0178 (0.004)* | 0.0094 (0.0037)* |
| School | 0.0361 (0.0093)* | 0.0291 (0.0079)* | 0.0099 (0.004)* | 0.0075 (0.0038)* |
| Stressful life events | 0.0054 (0.0035) | 0.0035 (0.0028) | 0.005 (0.0034) | 0.0096 (0.0045)* |
| General health | 0.0061 (0.0025)* | 0.0069 (0.0029)* | 0.0034 (0.0015)* | 0.0042 (0.0024) |
| Mental health | 0.0457 (0.0104)* | 0.042 (0.0096)* | 0.0198 (0.004)* | 0.0091 (0.0033)* |
| Physical health | 0.0425 (0.009)* | 0.0326 (0.0075)* | 0.011 (0.0042)* | 0.0113 (0.0041)* |
| Genetic | 0.0873 (0.051) | 0.0856 (0.0535) | 0.0217 (0.0618) | 0.0701 (0.0655) |
| Error | 0.4836 (0.0644)* | 0.5172 (0.0666)* | 0.6896 (0.0759)* | 0.7078 (0.0779)* |
| Covariance | 0.2083 | 0.2048 | 0.1938 | 0.1545 |
| Demographics ~ Friends and family | r=0.6727 (0.3038)* | r=0.4746 (0.3745) | r=0.5705 (0.2853)* | r=0.8026 (0.4357) |
| Demographics ~ Positive cognition | r=0.1986 (0.2577) | r=0.284 (0.2751) | r=0.758 (0.1325)* | r=0.8164 (0.2194)* |
| Demographics ~ School | r=-0.2519 (0.3072) | r=0.0786 (0.3716) | r=0.519 (0.2791) | r=0.5524 (0.3581) |
| Demographics ~ Stressful life events | r=0.2513 (0.5782) | r=-0.355 (0.7056) | r=0.4434 (0.4734) | r=0.856 (0.3916)* |
| Demographics ~ General health | r=0.9899 (0.2241)* | Out of bounds | Out of bounds | Out of bounds |
| Demographics ~ Mental health | r=0.6205 (0.2182)* | r=0.6914 (0.229)* | r=0.7722 (0.1182)* | r=0.7134 (0.198)* |
| Demographics ~ Physical health | r=0.5986 (0.2667)* | r=0.3393 (0.362) | r=0.6682 (0.3238)* | r=0.6974 (0.2869)* |
| Demographics ~ Genetic | r=0.5644 (0.1976)* | r=0.6702 (0.1958)* | r=0.6222 (0.1935)* | Out of bounds |
| Friends and family ~ Positive cognition | r=0.3519 (0.2066) | r=0.3847 (0.2108) | r=0.7095 (0.276)* | r=0.4557 (0.4778) |
| Friends and family ~ School | r=0.2535 (0.4198) | r=0.4022 (0.4337) | r=0.2977 (0.4819) | r=0.5625 (0.5552) |
| Friends and family ~ Stressful life events | r=0.5042 (0.3232) | r=0.8694 (0.2951)* | r=0.8953 (0.2516)* | r=-0.0918 (0.539) |
| Friends and family ~ General health | r=0.6191 (0.2083)* | r=0.684 (0.2051)* | r=0.4796 (0.1964)* | r=0.8385 (0.3917)* |
| Friends and family ~ Mental health | r=0.2921 (0.1849) | r=0.1858 (0.1957) | r=0.5411 (0.2537)* | r=0.6411 (0.3955) |
| Friends and family ~ Physical health | r=0.266 (0.1803) | r=0.3277 (0.1791) | r=0.6189 (0.2115)* | r=0.8568 (0.3076)* |
| Friends and family ~ Genetic | r=0.3019 (0.4191) | r=0.3644 (0.4053) | r=0.864 (0.4392)* | r=0.9426 (0.3518)* |
| Positive cognition ~ School | r=0.5663 (0.2232)* | r=0.5323 (0.2362)* | r=0.6391 (0.211)* | Out of bounds |
| Positive cognition ~ Stressful life events | r=0.4018 (0.1679)* | r=0.4245 (0.1642)* | r=0.7405 (0.1085)* | r=0.753 (0.2177)* |
| Positive cognition ~ General health | r=0.2351 (0.1807) | r=0.2306 (0.1817) | r=0.5733 (0.2186)* | r=0.5604 (0.2832)* |
| Positive cognition ~ Mental health | r=0.3855 (0.3739) | r=0.4753 (0.4298) | r=0.6018 (0.5646) | r=0.539 (0.4495) |
| Positive cognition ~ Physical health | r=0.337 (0.2735) | r=0.3803 (0.296) | r=0.6017 (0.3275) | r=0.7711 (0.3678)* |
| Positive cognition ~ Genetic | r=0.2945 (0.1872) | r=0.3289 (0.1873) | r=0.835 (0.1895)* | r=0.5779 (0.3096) |
| School ~ Stressful life events | r=0.2835 (0.1715) | r=0.3123 (0.1777) | r=0.5158 (0.2875) | r=0.7555 (0.335)* |
| School ~ General health | Out of bounds | Out of bounds | r=0.361 (0.596) | r=0.99 (0.4045)* |
| School ~ Mental health | r=0.3898 (0.422) | r=0.4053 (0.4519) | r=0.7553 (0.4217) | r=0.3722 (0.4799) |
| School ~ Physical health | r=0.5657 (0.402) | Out of bounds | r=0.0143 (0.6379) | r=0.6063 (0.3884) |
| School ~ Genetic | r=0.8995 (0.1628)* | r=0.8532 (0.1751)* | Out of bounds | Out of bounds |
| Stressful life events ~ General health | r=0.4473 (0.2771) | r=0.4373 (0.3193) | r=0.676 (0.3283)* | r=0.5025 (0.4123) |
| Stressful life events ~ Mental health | r=0.4906 (0.1802)* | r=0.5674 (0.175)* | r=0.5721 (0.2188)* | r=0.5777 (0.2815)* |
| Stressful life events ~ Physical health | r=-0.0187 (0.542) | r=0.2161 (0.6597) | r=-0.4455 (1.1255) | r=0.5741 (0.7231) |
| Stressful life events ~ Genetic | r=-0.322 (0.3476) | r=-0.5176 (0.3576) | r=-0.6531 (1.1526) | r=0.1944 (0.6511) |
| General health ~ Mental health | r=-0.4422 (0.401) | r=-0.4814 (0.402) | r=-0.3591 (0.9419) | r=0.0765 (0.6733) |
| General health ~ Physical health | r=-0.4891 (0.401) | r=-0.6585 (0.432) | Out of bounds | r=-0.6072 (0.7463) |
| General health ~ Genetic | r=-0.6484 (0.6619) | Out of bounds | r=-0.1423 (1.4276) | r=-0.0798 (0.7446) |
| Mental health ~ Physical health | r=0.1957 (0.5836) | r=-0.0723 (0.6208) | r=-0.468 (1.3237) | r=0.631 (0.7779) |
| Mental health ~ Genetic | r=-0.4119 (0.4294) | r=-0.5559 (0.4306) | r=-0.9096 (1.4562) | r=-0.3827 (0.6459) |
| Physical health ~ Genetic | r=-0.5052 (0.3297) | r=-0.4976 (0.3448) | Out of bounds | r=-0.1971 (0.5497) |
| Total variance explained | 0.5164 | 0.4828 | 0.3104 | 0.2922 |
| **Domains, GxD** $Y = X\beta+ g +sdem +sphy +smen+sgen+ssch+sfrf+spos+ssle +spos*g +... +\varepsilon$ | | | | |
| Demographics | 0.004 (0.0024) | 0.0028 (0.002) | 0.0119 (0.0058)* | 0.0128 (0.0061)* |
| Friends and family | 0.0203 (0.0064)* | 0.0232 (0.0073)* | 0.0177 (0.0065)* | 0.0019 (0.005) |
| Positive cognition | 0.1493 (0.0431)* | 0.1511 (0.0432)* | 0.0657 (0.0225)* | 0.018 (0.0077)* |
| School | 0.0493 (0.0129)* | 0.0409 (0.0113)* | 0.0185 (0.0054)* | 0.0058 (0.0045) |
| Stressful life events | 0.0022 (0.0019) | 0.001 (0.0016) | 0.0067 (0.0042) | 0.0059 (0.0055) |
| General health | 0.0101 (0.0044)* | 0.0101 (0.0049)* | 0.0057 (0.001)* | 0.0049 (0.0034) |
| Mental health | 0.0896 (0.0297)* | 0.0857 (0.0286)* | 0.0793 (0.0238)* | 0.0262 (0.0113)* |
| Physical health | 0.0431 (0.0103)* | 0.0319 (0.0084)* | 0.0093 (0.0044)* | 0.0109 (0.0056) |
| Genetic | 0.0334 (0.0398) | 0.029 (0.0434) | 0.001 (0.0507) | 0.1448 (0.0757) |
| Demographics x Genetic | Out of bounds | Out of bounds | 0.0235 (0.0154) | 0.0097 (0.0112) |
| Friends and family x Genetic | 0.0631 (0.0206)* | 0.0832 (0.0235)* | 0.0608 (0.0224)* | 0.0473 (0.0237)* |
| Mental health x Genetic | 0.105 (0.0225)* | 0.1031 (0.0232)* | 0.0587 (0.0217)* | 0.1116 (0.0274)* |
| Physical health x Genetic | 0.1346 (0.0252)* | 0.0969 (0.0244)* | 0.1287 (0.0273)* | 0.0353 (0.0244) |
| General health x Genetic | Out of bounds | Out of bounds | Out of bounds | Out of bounds |
| Positive cognition x Genetic | 0.1172 (0.0275)* | 0.098 (0.0274)* | 0.072 (0.025)* | Out of bounds |
| School x Genetic | 0.0753 (0.0239)* | 0.0887 (0.0259)* | 0.0474 (0.0185)* | 0.0287 (0.0204) |
| Stressful life events x Genetic | Out of bounds | Out of bounds | 0.0184 (0.0105) | 0.0705 (0.0223)* |
| Error | 0.1155 (0.0458)* | 0.1637 (0.0507)* | 0.3817 (0.061)* | 0.4738 (0.0823)* |
| Total variance explained | 0.8845 | 0.8363 | 0.6183 | 0.5262 |
| **Note.** Standardised variance component (standard error) for each depression symptom outcome (W1A, W1B, W2, W4). Correlation coefficients provided for models including covariance parameters. *indicates p value < 0.05. Out-of-bounds components are ones in which estimation resulted in negative variances, missing standard errors, or computed correlations that were +/-1. | | | | |

**Figure 2**. Gene-environment interactions for the domains model.

### 2B.1 Sensitivity analysis: Mental health domain removed

We re-computed the domain model (with environmental and genetic domains) with the mental health domain removed from the model. Total variance explained did not change from the model with all domains, but the contribution of the physical health domain substantially increased (**Table 14).**

| **Table 14.** Parameter values for domain influences on depression symptoms - mental health domains removed | | | | |
| --- | --- | --- | --- | --- |
| **Input** | **W1A** | **W1B** | **W2** | **W4** |
| **Domains** $Y = X\beta+ sdem +sphy +sgen+ssch+sfrf+spos+ssle +\varepsilon$ | | | | |
| Demographics | 0.0025 (0.002) | 0.0017 (0.0018) | 0.0126 (0.006)* | 0.0125 (0.0058)* |
| Friends and family | 0.0298 (0.0077)* | 0.0329 (0.0083)* | 0.0242 (0.0078)* | 0.0094 (0.0056) |
| Positive cognition | 0.182 (0.0467)* | 0.1747 (0.0454)* | 0.0818 (0.0248)* | 0.0218 (0.0086)* |
| School | 0.065 (0.0161)* | 0.0558 (0.0144)* | 0.0239 (0.0082)* | 0.0104 (0.0056) |
| Stressful life events | 0.0054 (0.0033) | 0.0017 (0.0024) | 0.0097 (0.0048)* | 0.0127 (0.0058)* |
| General health | 0.0144 (0.0063)* | 0.0142 (0.0062)* | 0.0122 (0.006)* | 0.0081 (0.0046) |
| Physical health | 0.1178 (0.0208)* | 0.1011 (0.0185)* | 0.0505 (0.0113)* | 0.0287 (0.0081)* |
| Genetic | 0.0587 (0.0543) | 0.0597 (0.0578) | 0.0451 (0.0731) | 0.1521 (0.0842) |
| Error | 0.5244 (0.0633)* | 0.5582 (0.0664)* | 0.74 (0.0766)* | 0.7443 (0.0849)* |
| Total variance explained | 0.4756 | 0.4418 | 0.26 | 0.2557 |
| **Note.** Standardised variance component (standard error) for each depression symptom outcome (W1A, W1B, W2, W4); *indicates p value < 0.05. Out-of-bounds components are ones in which estimation resulted in negative variances, missing standard errors, or computed correlations that were +/-1. | | | | |

## *2C. System-specific influences on depression symptoms*

| **Table 15.** Parameter values for system influences on depression symptoms | | | | |
| --- | --- | --- | --- | --- |
| **Input** | **W1A** | **W1B** | **W2** | **W4** |
| **Systems** $Y = X\beta+biosystem +psychsystem +social+built +natural +\varepsilon$ | | | | |
| Genetic/Biological systems | 0.0474 (0.0453) | 0.0473 (0.0493) | Out of bounds | 0.1664 (0.0843)* |
| Psychological systems | 0.4548 (0.0302)* | 0.4272 (0.0299)* | 0.2263 (0.0237)* | 0.0793 (0.013)* |
| Social environment | 0.0646 (0.0109)* | 0.0538 (0.01)* | 0.0674 (0.0123)* | 0.04 (0.0102)* |
| Built environment | 0.0014 (0.0016) | 0.0015 (0.0017) | 0.0065 (0.0032)* | 0.0053 (0.0034) |
| Natural environment | 0.001 (0.0012) | 6e-04 (0.0012) | Out of bounds | 0.0012 (0.0021) |
| Error | 0.4309 (0.0508)* | 0.4696 (0.0546)* | 0.7287 (0.0689)* | 0.7079 (0.0849)* |
| Total variance explained | 0.5691 | 0.5304 | 0.2713 | 0.2921 |
| **Systems, rS** $Y = X\beta+biosystem+psychsystem +social+built +natural +\varepsilon, \sigma_{ss}⧣ 0$ | | | | |
| Genetic/Biological systems | 0.0607 (0.0467) | 0.0588 (0.05) | Out of bounds | 0.1737 (0.0847)* |
| Psychological systems | 0.4645 (0.05)* | 0.4315 (0.0465)* | 0.2435 (0.0309)* | 0.0893 (0.0155)* |
| Social environment | 0.07 (0.0114)* | 0.0594 (0.0104)* | 0.0677 (0.0122)* | 0.0364 (0.0098)* |
| Built environment | 9e-04 (0.0015) | 0.001 (0.0016) | 0.0061 (0.0031) | 0.0051 (0.0033) |
| Natural environment | 0.0013 (0.0013) | 0.001 (0.0012) | Out of bounds | 0.0011 (0.002) |
| Error | 0.4439 (0.0623)* | 0.4741 (0.0656)* | 0.7367 (0.0834)* | 0.7027 (0.091)* |
| Covariance | -0.0412 | -0.0256 | -0.0305 | -0.0084 |
| Genetic ~ Psychological | r=-0.5973 (0.3007)* | r=-0.5341 (0.3065) | Out of bounds | r=-0.1995 (0.2083) |
| Psychological ~ Social | r=0.4417 (0.0938)* | r=0.4516 (0.0989)* | r=0.4011 (0.1116)* | r=0.3623 (0.1699)* |
| Total variance explained | 0.5561 | 0.5259 | 0.2633 | 0.2973 |
| **Systems, SxS** $Y = X\beta+biosystem+psychsystem+social+built+natural+social*built+ ... +\varepsilon$ | | | | |
| Genetic/Biological systems | 0.0308 (0.0349) | 0.0241 (0.0388) | Out of bounds | 0.1594 (0.0789)* |
| Psychological systems | 0.3981 (0.0314)* | 0.3791 (0.0309)* | 0.2026 (0.0238)* | 0.0739 (0.0134)* |
| Social environment | 0.0617 (0.0107)* | 0.0548 (0.0104)* | 0.0633 (0.0125)* | 0.0306 (0.0102)* |
| Built environment | 8e-04 (0.0012) | 8e-04 (0.0013) | 0.0047 (0.0026) | 0.0047 (0.0031) |
| Natural environment | 8e-04 (9e-04) | 7e-04 (0.001) | Out of bounds | 0.0013 (0.002) |
| Genetic x Psychological | 0.2996 (0.0323)* | 0.2619 (0.0324)* | 0.2348 (0.0347)* | 0.1206 (0.0329)* |
| Genetic x Social | 0.0825 (0.0233)* | 0.0991 (0.0254)* | 0.1093 (0.0296)* | 0.095 (0.0324)* |
| Genetic x Built | 0.0237 (0.014) | 0.0152 (0.0151) | 0.0419 (0.0236) | Out of bounds |
| Error | 0.1019 (0.0408)* | 0.1644 (0.0463)* | 0.3564 (0.0668)* | 0.5186 (0.0852)* |
| Total variance explained | 0.8981 | 0.8356 | 0.6436 | 0.4814 |
| **Systems, rS sig SxS** $Y = X\beta+biosystem+psychsystem+social+built+natural+social*built+ ... +\varepsilon$ $\sigma_{ss}⧣ 0$ | | | | |
| Genetic/Biological systems | 0.0455 (0.0351) | 0.0392 (0.0416) | Out of bounds | 0.1795 (0.085)* |
| Psychological systems | 0.3762 (0.0406)* | 0.3846 (0.0438)* | 0.2192 (0.0315)* | 0.0772 (0.0156)* |
| Social environment | 0.0589 (0.0101)* | 0.0601 (0.0119)* | 0.0753 (0.0156)* | 0.0362 (0.0122)* |
| Built environment | 7e-04 (0.0011) | 7e-04 (0.0013) | 0.0056 (0.0029) | 0.0046 (0.0032) |
| Natural environment | 7e-04 (9e-04) | 7e-04 (0.001) | Out of bounds | 0.0011 (0.002) |
| Genetic x Psychological | 0.2884 (0.0364)* | 0.2674 (0.0381)* | 0.2514 (0.041)* | 0.1241 (0.0351)* |
| Genetic x Social | 0.0856 (0.0238)* | 0.1014 (0.0272)* | 0.118 (0.0333)* | 0.096 (0.0343)* |
| Error | 0.1144 (0.0402)* | 0.1954 (0.0502)* | 0.4528 (0.078)* | 0.5503 (0.0957)* |
| Covariance | 0.0296 | -0.0494 | -0.1184 | -0.069 |
| Genetic ~ Psychological | r=-0.4795 (0.286) | r=-0.5591 (0.384) | Out of bounds | r=-0.2357 (0.2272) |
| Genetic ~ Social | r=0.1402 (0.1349) | r=-0.6332 (0.4486) | Out of bounds | r=-0.3736 (0.2734) |
| Psychological ~ Social | r=0.4721 (0.0959)* | r=0.4911 (0.0978)* | r=0.4995 (0.1064)* | r=0.4423 (0.1822)* |
| Total variance explained | 0.8856 | 0.8046 | 0.5472 | 0.4497 |
| **Note.** Standardised variance component (standard error) for each depression symptom outcome (W1A, W1B, W2, W4); *indicates p value < 0.05. Out-of-bounds components are ones in which estimation resulted in negative variances, missing standard errors, or computed correlations that were +/-1. | | | | |

### 2C.1 Sensitivity analysis: Reduced systems models

We conducted a sensitivity analysis where we removed the psychological system from the model (and kept genetic-social and social-built interactions and covariances). Then we removed both psychological and social systems, and finally, the psychological, social, and genetic systems (**Table 16**). We found that when psychological systems were removed, the variance explained by social systems substantially increased. When psychological and social systems were removed, the variance was allocated to genetic systems. When all three are removed, the Built and Natural systems still explain little variance in depression symptoms.

| **Table 16.** Parameter values for system influences on depression symptoms | | | | |
| --- | --- | --- | --- | --- |
| **Input** | **W1A** | **W1B** | **W2** | **W4** |
| **Systems (no psych), rS** $Y = X\beta+bio +social+built +natural +\varepsilon, \sigma_{ss}⧣ 0$ | | | | |
| Genetic/Biological systems | 0.0622 (0.0399) | 0.0819 (0.0522) | 0.0511 (0.0676) | 0.1728 (0.0865)* |
| Social environment | 0.2586 (0.0202)* | 0.1774 (0.0137)* | 0.1574 (0.0161)* | 0.0791 (0.0147)* |
| Built environment | 1e-04 (0.0011) | 0.0019 (0.0019) | 0.005 (0.0031) | 0.0036 (0.003) |
| Natural environment | 0.002 (0.0014) | 0.0045 (0.0026) | 0.0026 (0.0024) | 0.0011 (0.002) |
| Genetic x Social | 0.4897 (0.043)* | 0.3526 (0.0366)* | 0.2813 (0.0389)* | 0.1707 (0.0389)* |
| Social x Built | 0.0068 (0.0088) | Out of bounds | Out of bounds | 0.0052 (0.0106) |
| Error | 0.119 (0.0451)* | 0.2843 (0.0603)* | 0.5135 (0.0799)* | 0.6239 (0.0957)* |
| Covariance | 0.0616 | 0.1005 | -0.006 | -0.0563 |
| Genetic ~ Social | r=-0.1796 (0.216) | r=0.0599 (0.1853) | r=-0.284 (0.3357) | r=-0.3362 (0.2293) |
| Social ~ Built | Out of bounds | Out of bounds | r=0.3437 (0.2579) | r=0.5434 (0.4137) |
| Total variance explained | 0.881 | 0.7157 | 0.4865 | 0.3761 |
| **Systems (no psych or soc)** $Y = X\beta+bio+built+natural +\varepsilon$ | | | | |
| Genetic/Biological systems | 0.2612 (0.0923)* | 0.2592 (0.0913)* | Out of bounds | 0.2074 (0.0923)* |
| Built environment | 0.0026 (0.0029) | 0.0026 (0.0029) | Out of bounds | 0.0071 (0.004) |
| Natural environment | 0.0033 (0.0028) | 0.0033 (0.0028) | Out of bounds | 0.0037 (0.0031) |
| Error | 0.7329 (0.0924)* | 0.735 (0.0914)* | Out of bounds | 0.7818 (0.0923)* |
| Total variance explained | 0.2671 | 0.265 | Out of bounds | 0.2182 |
| **Systems (no psych, soc, or gen)** $Y = X\beta+built+natural +\varepsilon$ | | | | |
| Built environment | 0.0024 (0.0028) | 0.0023 (0.0028) | Out of bounds | 0.0071 (0.004) |
| Natural environment | 0.0034 (0.0028) | 0.0035 (0.0029) | Out of bounds | 0.0039 (0.0031) |
| Error | 0.9942 (0.0042)* | 0.9942 (0.0042)* | Out of bounds | 0.989 (0.0052)* |
| Total variance explained | 0.0058 | 0.0058 | Out of bounds | 0.011 |
| **Note.** Standardised variance component (standard error) for each depression symptom outcome (W1A, W1B, W2, W4); *indicates p value < 0.05. Out-of-bounds components are ones in which estimation resulted in negative variances, missing standard errors, or computed correlations that were +/-1. | | | | |
